# The Degradation of Synthetic Polymeric Scaffolds With Strut-like Architecture Influences the Mechanics-dependent Repair Process of an Osteochondral Defect *in Silico*

**DOI:** 10.3389/fbioe.2022.846665

**Published:** 2022-03-10

**Authors:** Martina Tortorici, Ansgar Petersen, Georg N. Duda, Sara Checa

**Affiliations:** Julius Wolff Institute, Berlin Institute of Health at Charité–Universitätsmedizin Berlin, Berlin, Germany

**Keywords:** osteochondral defect, tissue engineering, scaffold degradation, computer model, mechanobiolgy

## Abstract

Current clinical treatments of osteochondral defects in articulating joints are frequently not successful in restoring articular surfaces. Novel scaffold-based tissue engineering strategies may help to improve current treatment options and foster a true regeneration of articulating structures. A frequently desired property of scaffolds is their ability to degrade over time and allow a full restoration of tissue and function. However, it remains largely unknown how scaffold degradation influences the mechanical stability of the tissue in a defect region and, in turn, the regenerative process. Such differing goals–supporting regeneration by degrading its own structure–can hardly be analyzed for tissue engineered constructs in clinical trials and *in vivo* preclinical experiments. Using an *in silico* analysis, we investigated the degradation-induced modifications in material and architectural properties of a scaffold with strut-like architecture over the healing course and their influence on the mechanics-dependent tissue formation in osteochondral defects. The repair outcome greatly varied depending on the degradation modality, i.e. surface erosion or bulk degradation with and without autocatalysis, and of the degradation speed, i.e. faster, equal or slower than the expected repair time. Bulk degradation with autocatalysis, independently of degradation speed, caused the mechanical failure of the scaffold prior to osteochondral defect repair and was thereby deemed inappropriate for further application. On the other hand, scaffolds with strut-like architecture degrading by both surface erosion and bulk degradation with slow degradation speed resulted in comparably good repair outcomes, thereby indicating such degradation modalities as favorable for the application in osteochondral defects.

## Introduction

Osteochondral defects affect the articular cartilage and the subchondral bone and are frequently a result of traumatic events or degenerative processes (Davis et al., 2021). As cartilage presents limited natural regenerative potential, the pre-injury tissue configuration is rarely restored by spontaneous repair processes (Hunziker et al., 2015). Osteochondral defects are associated with relevant pain and limit the joint function and mobility of patients. Additionally, they might initiate degenerative processes in surrounding tissues, eventually leading to further degeneration of the complete joint (Hunziker et al., 2015). To stop the degenerative progression of osteochondral defects, a timely and effective treatment is of great importance. However, current clinical treatments are unable to restore healthy articulation or are associated with severe limitations (Davis et al., 2021), such as the need for multiple interventions or a triggering of degeneration in previously unaffected cartilage areas (Nukavarapu & Dorcemus, 2013; Hunziker et al., 2015). The development of scaffold-based tissue engineering (TE) strategies has the potential of supporting osteochondral defect healing, thereby complementing current clinical treatment options with a truly regenerative strategy.

The regeneration of osteochondral defects might be achieved by TE scaffolds imparting specific mechanical cues to guide the differentiation of mesenchymal stromal cells (MSCs) (Davis et al., 2021). In fact, numerous *in vitro* evaluations have shown that MSCs can sense and respond to mechanical stimulation (Delaine-Smith & Reilly, 2012; Schreivogel et al., 2019). Moreover, computational models employing mechano-biological rules to determine tissue formation have highlighted the importance of mechanics on the healing process of osteochondral defects. For example, the repair of osteochondral defects in minipigs was reproduced by describing tissue formation under specific ranges of minimum principal strain (Duda et al., 2005). Moreover, typical features of the *in vivo* repair pattern were obtained simulating tissue formation based on a mechanical stimulus, which was calculated from octahedral shear strain and fluid velocity (Kelly & Prendergast, 2005; Tortorici et al., 2021). To support the mechanics-dependent healing of osteochondral defects, scaffolds with different mechanical properties have been compared *in vivo*. For example, poly (lactide-co-glycolide) (PLGA) scaffolds with high and low stiffness were implanted in osteochondral defects in sheep, resulting in better subchondral bone formation with the stiffer scaffolds, although without improvements of cartilage formation (Schlichting et al., 2008). In another case, three PLGA scaffolds with different elastic moduli were tested in osteochondral defects in rabbit, observing that the two scaffolds with lower elastic moduli better supported the repair process compared to the stiffer one (Ikeda et al., 2009). In both cited examples, however, the different mechanical properties of the scaffolds were achieved by different scaffold porosities. Therefore, a clear distinction of mechanical stimuli from morphological cues (e.g., pore size) was not possible. To separately evaluate the influence of scaffold stiffness and morphology on osteochondral defect repair, an *in silico* model was recently developed (Tortorici et al., 2021). By investigating *in silico* the mechanical behavior of simple scaffold designs upon implantation into an osteochondral defect, it was possible to suggest scaffold properties that are supportive for the treatment of osteochondral defects. In fact, the computational results indicated that a scaffold with material elastic modulus in the low GPa range and an architecture reducing both compressive and radial displacements has the potential to improve osteochondral defect repair compared to an untreated defect (Tortorici et al., 2021). However, only non-degradable scaffolds were investigated in this model.

The ability to degrade in the environment of the body (see Table 1 for definitions) is often listed amongst the properties of an ideal scaffold or material for TE (Bose et al., 2012; Middleton & Tipton, 2000; Turnbull et al., 2018). In fact, a biodegradable material has the advantage of not requiring a second surgical intervention for its removal (Casalini, 2017), while avoiding concerns on its long term influence at the site of implantation, e.g., the elicitation of a foreign body response (Griffith, 2002) or the stress shielding of the adjacent tissues (Muschler et al., 2004). Material degradation can be even employed for the timely delivery of specific chemical cues or drugs (Casalini, 2017). However, the process of degradation may result in modifications of the chemical, morphological, biological, and mechanical properties of a material (Nair & Laurencin, 2007), and thereby needs to be carefully tuned for the individual application. Synthetic biocompatible and biodegradable polymers (see Table 1 for definitions) typically degrade by hydrolysis (Middleton & Tipton, 2000), i.e., by a shortening of the polymeric chains caused by a reaction with water molecules (Casalini, 2017). Depending on degradation kinetic and small molecule diffusion, two types of hydrolytic degradation are possible: surface erosion or bulk degradation (Casalini, 2017). In surface erosion, the hydrolytic reaction is faster than water diffusion into the material, resulting in a thinning of the scaffold features without a reduction in molecular weight (Middleton & Tipton, 2000; Woodruff & Hutmacher, 2010). Surface erosion is typically observed in polyanhydrides and polyorthoesters (Nair & Laurencin, 2007). In bulk degradation, the hydrolytic reaction is slower than water diffusion into the material, resulting in a uniform reduction of molecular weight in the scaffold without weight loss nor volume modifications (Woodruff & Hutmacher, 2010; Casalini, 2017). Polyesters are known to degrade by bulk degradation with acidic degradation by-products (Casalini, 2017). If the production of the acidic degradation by-products is faster than their diffusion away from the material, these acidic by-products speed up the reduction in polymeric molecular weight in the bulk of the device (Middleton & Tipton, 2000), a phenomenon known as autocatalysis. The establishment of autocatalysis depends not only on polymer chemistry, but also on architectural features of the device, e.g., its thickness (Grizzi et al., 1995). Bulk degradation with autocatalysis has been observed *in vivo* in devices made from poly (lactide-co-glycolide) (PLGA) and poly (lactic acid) (PLA) (Therin et al., 1992), but not from poly (ε-caprolactone) (PCL) (Woodruff & Hutmacher, 2010). The experimentally observed degradation behaviors of some of the polymers that are commonly investigated for TE applications are summarized in Table 2.

**TABLE 1:** Definitions of degradation-related terms.

Property	Definition	References
Degradation	“Chain scission process during which polymer chains are cleaved to form oligomers and finally to form monomers”	Göpferich, (1996)
Erosion	“Loss of material owing to monomers and oligomers leaving the polymer”	Göpferich, (1996)
Biodegradable	“Solid polymeric devices which break down to macromolecule degradation with dispersion in an animal body but no proof for elimination from the body”	Vert et al. (1992)
Bioresorbable	“Solid materials which can degrade and further resorb *in vivo*, i.e. which are eliminated through natural pathways either because of simple filtration of degradation by-products or after their metabolization”	Vert et al. (1992)

**TABLE 2:** Degradation behaviors of some of the polymers that are commonly investigated in tissue engineering.

Polymer	Abbreviation	Degradation modality	Degradation time
Poly (carboxyphenoxypropane-sebacic acid)	P(CPP-SA)	Surface degradation (Tamada & Langer, 1993)	6–8 weeks (Katti et al., 2002)
Poly (ε-caprolactone)	PCL	Bulk degradation without autocatalysis (Lam et al., 2008)	2–4 years, depending on molecular weight (Woodruff & Hutmacher, 2010)
Poly (D,L-lactic acid)	PDLLA	Bulk degradation with autocatalysis (Therin et al., 1992)	12–16 months (Nair & Laurencin, 2007)
Poly (lactide-co-glycolide)	PLGA	Bulk degradation (Tamada & Langer, 1993) with autocatalysis (Therin et al., 1992)	1–6 months, depending on co-polymer ratio (Nair & Laurencin, 2007)

As architecture is, next to the material type, the most important factor determining the mechanical properties of a scaffold, e.g., its compressive modulus (Marchiori et al., 2019), modifications in mechanical behavior can be expected as a consequence of a surface erosion process. Moreover, the molecular weight of polymers is closely related to their mechanical properties (Nunes et al., 1982). Consequently, a reduction in molecular weight resulting from a bulk degradation process (with or without autocatalysis) may modify the mechanical properties of a scaffold, as previously observed experimentally, e.g., in PLGA (Wu & Ding, 2004) and PCL (Lam et al., 2008) scaffolds. Given the link between scaffold-dependent mechanical cues and tissue formation discussed above, it is important to evaluate how modifications in scaffold mechanical properties due to degradation would influence the healing process. However, an experimental investigation of this phenomenon presents significant technical challenges, such as the need for long term *in vitro* and *in vivo* experiments (Pan, 2015). Computational models can offer first evaluations in a simplified environment, providing indications for future, more complex experimental tests. A number of computational studies have already explored tissue growth in presence of a degradable scaffold in the context of bone formation (Adachi et al., 2006; Chen et al., 2011; Gorriz et al., 2015). To the best of our knowledge, such an evaluation is so far lacking for osteochondral defects.

Here, we employed a previously developed computational model of scaffold-supported osteochondral defect repair (Tortorici et al., 2021) to study the consequences of scaffold degradation on the mechanics-dependent repair process. To do so, an “artificial” scaffold with strut-like architecture was modelled, whose material and architectural properties prior to degradation were those of the simplest design fostering an improved osteochondral defect repair compared to the untreated case, as previously established (Tortorici et al., 2021). The scaffold was defined “artificial” because its axisymmetric architecture and its material properties do not model a specific scaffold nor material currently investigated for tissue engineering. Three types of polymeric scaffold degradation, i.e. surface erosion, bulk degradation, and bulk degradation with autocatalysis, as well as different degradation rates were studied. Although ceramic (Marques et al., 2021) and metallic (Lv et al., 2021) degradable scaffolds have also been developed, their degradation modalities are different from the ones of polymeric materials and were not investigated here.

## Materials and Methods

An iterative computational model simulated the mechanics-dependent tissue formation within an osteochondral defect implanted with a scaffold in a knee femoral condyle. The model represented a focal osteochondral defect resulting from surgical intervention to treat damaged chondral or bone tissue, e.g., in consequence of trauma, early osteoarthritis or disease-related bone lesions. Therefore, the tissues outside the defect were simulated as healthy. A detailed description of the model has been provided elsewhere (Tortorici et al., 2021) and is here briefly summarized. Amongst the scaffolds evaluated in the cited model, the scaffold with the simplest architecture that resulted in improved repair compared to the untreated osteochondral defect was selected for the present investigation. As an additional feature compared to the previously published model, scaffold degradation was implemented here, as described in the following sections.

### Model of Osteochondral Defect Repair With Scaffold

**FIGURE 1:**
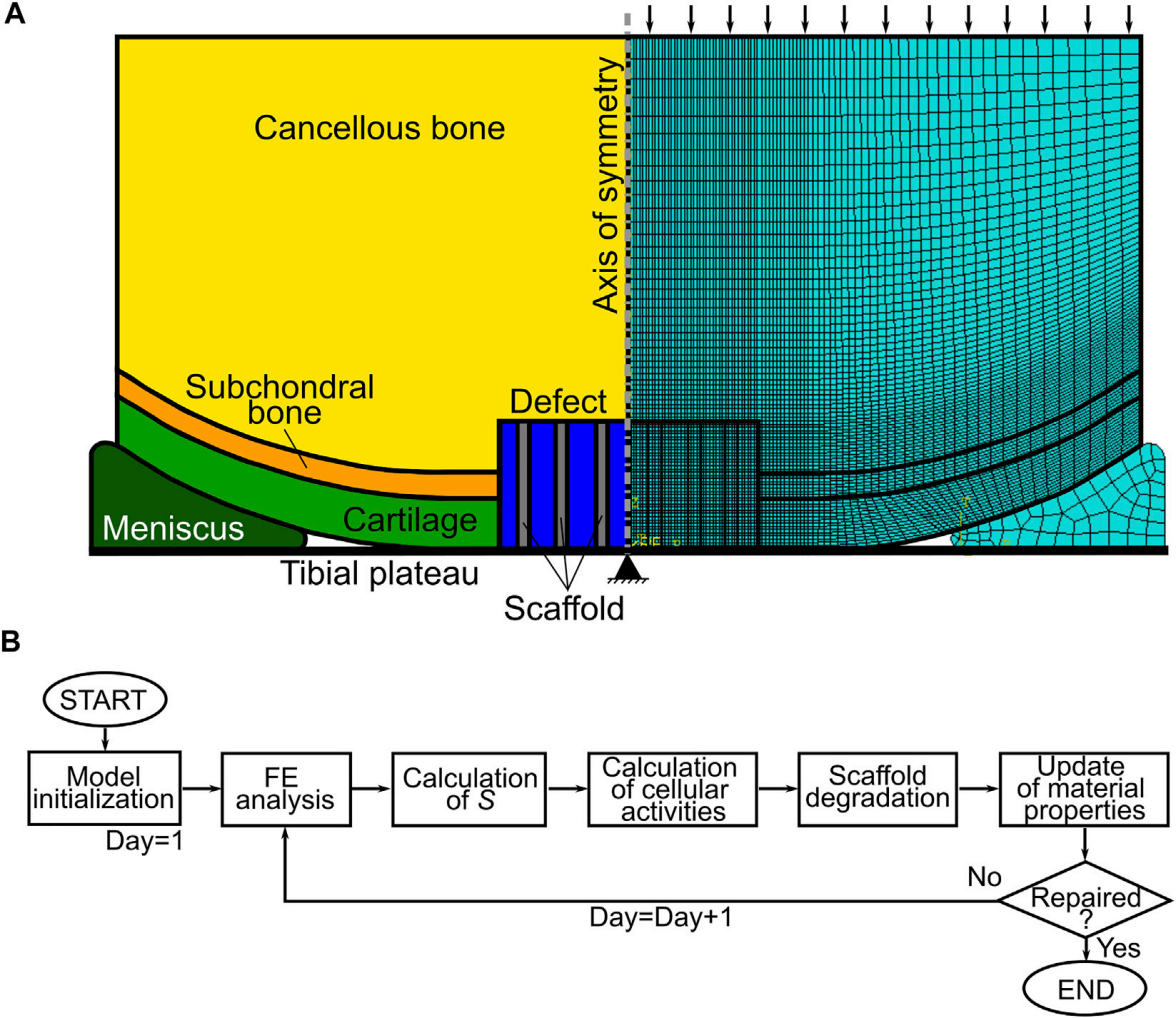
Model of osteochondral defect repair. **(A)** Geometry (left) and mesh (right) of the axisymmetric model of knee femoral condyle. Black arrows and the black triangle show the applied pressure and the encastre boundary condition in the finite element (FE) analysis, respectively; **(B)** workflow of the model. *S* = mechanics-dependent stimulus of tissue formation.

A simplified axisymmetric geometry of a knee femoral condyle laying on a meniscus and a tibial plateau was built in a finite element (FE) solver (Abaqus, Dassault Système) (Figure 1A). The femoral condyle was composed of healthy cartilage, subchondral bone, cancellous bone, and an osteochondral defect with a radius and a depth of 5 mm. Moreover, a scaffold composed of three vertical struts (thickness of 0.5 mm) was modelled in the defect. The scaffold material had the following properties: porosity of 50%; elastic modulus of 1,000 MPa; permeability of 3.63 × 10^–8^ mm/s; void ratio of 4; bulk modulus of grain of 0 MPa. Moreover, cells could diffuse through the scaffold material with a diffusion coefficient of 0.01 mm^2^/day. At the beginning of the simulation, the areas of the osteochondral defect that were not occupied by the scaffold were filled with granulation tissue. All biological tissues were modelled as isotropic and poroelastic, except healthy cartilage, which was isotropic and hyperelastic. However, newly formed cartilage within the osteochondral defect was isotropic and poroelastic, as later described. In addition, the meniscus was modelled as transversally isotropic and poroelastic. The values assigned to the material properties are listed in Table 3. The tibial plateau was modelled as a rigid wire.

**TABLE 3:** Properties of biological tissues (Kelly & Prendergast, 2005; Chen et al., 2011; Tortorici et al., 2021). All poroelastic tissues had a specific weight of the wetting liquid of 9.74 × 10^–6^ N/mm^3^ and a bulk modulus of fluid of 2,300 MPa. The axial, radial, and circumferential directions are indicated by 1, 2, and 3, respectively. *E*: elastic modulus; *ν*: Poisson’s ratio; *G*: shear modulus.

Isotropic and poroelastic tissues							
Tissue	Diffusion coefficient (mm^2^/day)	Elastic modulus (MPa)	Poisson’s ratio	Permeability (mm/s)	Void ratio	Bulk modulus of grain (MPa)	
Subchondral bone	0.01	17,000	0.3	9.74 × 10^–11^	0.042	13,920	
Cancellous bone	0.01	6,000	0.3	3.63 × 10^–8^	4	13,920	
Poroelastic cartilage	0.05	10	0.167	4.87 × 10^–8^	4	3,700	
Fibrous tissue	0.10	2	0.167	9.74 × 10^–8^	4	2,300	
Granulation tissue	0.80	0.2	0.167	9.74 × 10^–8^	4	2,300	
**Transversally Isotropic and poroelastic tissue**							
**Tissue**	**Elastic modulus (MPa)**		**Poisson’s ratio**	**Shear modulus (MPa)**	**Permeability (mm/s)**	**Void ratio**	**Bulk modulus of grain (MPa)**
Meniscus	• *E_1_* = 0.5		• *ν_12_* = 0.5	• *G_12_* = 0.167	4.87 × 10^–8^	4	3,700
	• *E_2_* = 0.5		• *ν_23_* = *ν_31_* = 0.0015	• *G_23_* = *G_31_* = 0.05			
	• *E_3_* = 100						
**Isotropic and hyperelastic tissue**							
**Tissue**	**Strain energy potential**		**C10 (MPa)**	**D1 (MPa)**	**Permeability (mm/s)**		
Hyperelastic cartilage	Neo-Hookean		2.14	0.399	4.87 × 10–8		

The femoral condyle-tibial plateau, femoral condyle-meniscus, and meniscus-tibial plateau contacts were modelled as “hard contact” in the normal direction and as frictionless in the tangential one. The model was meshed with elements type CAX8RP. Specifically, the osteochondral defect was meshed with 1,600 elements having a seed size of 0.125 mm, while a coarser mesh with seed size up to 0.8 mm was used in the rest of the model (Figure 1A right).

The model underwent a 1 s compression step, followed by a 0.5 s consolidation step. The load was applied on the upper surface of the cancellous bone in the form of a 0.637 MPa pressure (Tortorici et al., 2021). All displacement and rotation degrees of freedom were restrained in the tibial plateau at the axis of symmetry (Figure 1A). The following initial conditions were implemented in the whole model: pore pressure of 0 MPa; and saturation of 1 mm^3^/mm^3^. During the consolidation step, a pore pressure of 0 MPa was set at the free cartilage edges.

The octahedral shear strain (γ) and the fluid velocity (v) in the defect were used to compute a mechanical stimulus (*S*) as indicated by Equation 1:

$$S=\frac{\gamma }{a}+\frac{v}{b}$$ (1)

Where *a* = 3.75% and *b* = 3 × 10^–3^ mm/s were empirical constants (Kelly & Prendergast, 2005). Tissue formation was described by thresholds of *S*: bone resorption if 0 ≤ *S* < 0.01; bone formation if 0.01 ≤ *S* < 1; cartilage formation if 1 ≤ *S* < 3; fibrous tissue formation if *S* ≥ 3.

The mechanics-dependent cellular activities, specifically mitosis, apoptosis, and MSCs differentiation, were simulated based on *S* using a Matlab (MathWorks) script. The defect was represented by a 40 x 40 elements matrix, with elements corresponding to the ones in the FE mesh. Each element could be occupied by a maximum of 100 cells. Cells could also populate elements belonging to the struts of the scaffold due to the porosity of the scaffold material. In addition to MSCs, three cellular phenotypes were simulated: osteoblasts, chondrocytes, and fibroblasts, corresponding to bone, cartilage, and fibrous tissue, respectively. If the value of *S* in an element was within the thresholds fostering the formation of a tissue, 5% of the MSCs differentiated into the cell phenotype of that specific tissue. Moreover, the already existing cells of the tissue performed mitosis by increasing of 5%, while all other cell phenotypes underwent apoptosis by decreasing of 15% in number. MSCs had a mechanics-independent mitosis rate of 15%. The mechanics-dependent bone resorption was simulated by a 10% reduction in the number of osteoblasts.

At the initial time, the elements neighboring cancellous bone were completely filled with MSCs, while the elements in the rest of the defect were empty of cells. MSCs migration, simulated as a diffusion process, progressively populated the whole defect with cells. The diffusion process was modelled by solving a mass diffusion problem in a second FE model, where only the defect region was implemented and meshed with 1,600 elements type DC2D4.

It was assumed that each cell phenotype would produce the corresponding tissue proportionally to its number. Therefore, material properties in the defect changed as a consequence of cellular activities and needed to be updated at every iteration of the model (Figure 1B). For each element, the elastic modulus, the Poisson’s ratio, the permeability, the bulk modulus of grain and the diffusion coefficient were calculated as the weighted average of the materials occupying the element itself, as indicated by Equation 2:

$$X=\frac{1}{N_{MAX}}\left[\left(N_{MAX}-\sum_{i=1}^{n_{t}}N_{i}\right)\cdot X_{Gran}+\sum_{i=1}^{n_{t}}X_{i}\cdot N_{i}\right]$$ (2)

Where *X* is one of the parameters listed above, *N*_*MAX*_ is the maximum number of cells allowed in each element, *n*_*t*_ indicates the species (granulation tissue, bone, cartilage, fibrous tissue, and scaffold), *N* is the volume fraction occupied by the species, and *X*_*Gran*_ is the value that the parameter assumes for granulation tissue. Moreover, the properties of the scaffold (specifically, elastic modulus and volume fraction) varied in consequence of its degradation, as described in detail in the following section.

After the update of the material properties in the defect region, a new FE analysis of the femoral condyle begun, marking the beginning of a new iteration (Figure 1B). One iteration roughly corresponded to 1 day of the *in vivo* repair process. For untreated osteochondral defects and osteochondral defects implanted with non-degradable scaffolds, repair was calculated to be achieved at day 50 (Tortorici et al., 2021). To enable all models implementing degradable scaffolds to reach the equilibrium state, i.e. the absence of variations in tissue distributions, the simulations in this work were run until day 125, i.e. until 25 days after the expected time of complete degradation of the scaffold with the lowest degradation speed (see following section).

### Model of Polymeric Scaffold Degradation

**FIGURE 2:**
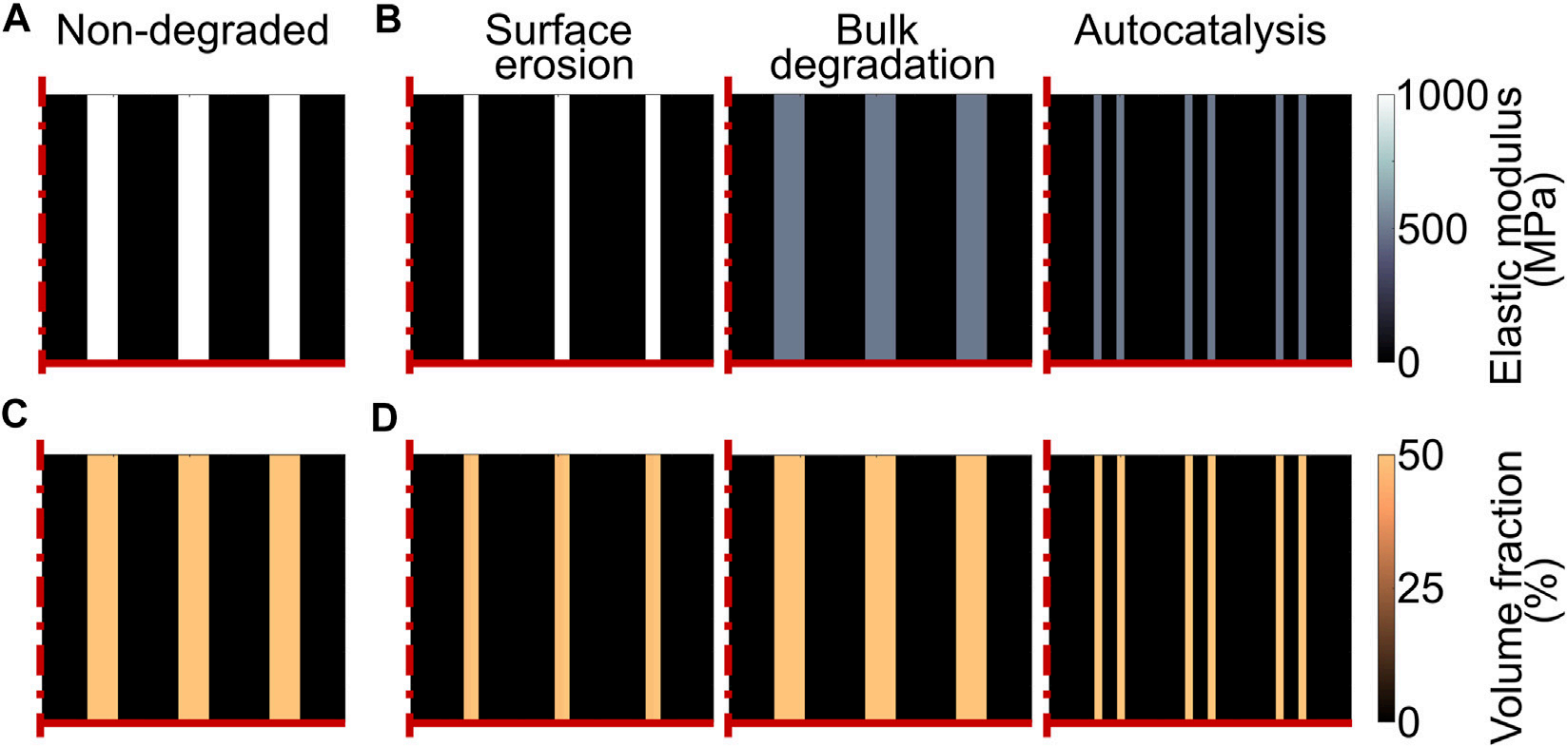
Model of scaffold degradation in osteochondral defect. **(A)** and **(C)** Elastic modulus and volume fraction, respectively, of the scaffold material prior to degradation; **(B)** and **(D)** Elastic modulus and volume fraction, respectively, of the “artificial” scaffold with matched degradation speed at day 25 (complete scaffold degradation set to day 50). The left, middle, and right columns show the scaffold degrading by surface erosion, bulk degradation, and bulk degradation with autocatalysis, respectively. At the time point shown in the plots, the surface and bulk elements of the scaffold degrading by surface erosion and bulk degradation with autocatalysis, respectively, were completely degraded. The dash-dot and solid red lines mark the axis of symmetry and the articular interface, respectively. The legends to interpret the plots are on the right side of the corresponding rows. The volume fraction is expressed as percentage of the total volume in one element.

The degradation of the scaffold material was modelled by varying the properties of the scaffold as a function of time, i.e. as a function of the model iteration. Three types of polymeric scaffold degradation by hydrolysis were modelled, i.e. surface erosion, bulk degradation, and bulk degradation with autocatalysis. Experimentally, the degradation of a scaffold by one of these three modalities is determined by both the chemistry of the material and the architecture of the device (von Burkersroda et al., 2002). Thus, scaffolds degrading by different modalities would most likely have different properties in their non-degraded state. To evaluate whether one of these degradation modalities was particularly advantageous or disadvantageous in supporting the repair of osteochondral defects, an “artificial” scaffold with strut-like architecture was investigated (Figures 2A,C), having the same material properties and architectural features prior to the onset of degradation, but the possibility to degrade by each one of the three modalities in turn. When comparing the three different degradation modalities, a linear degradation behavior was employed. Moreover, the influence of the degradation rate was evaluated by defining three speeds: fast, matched, and slow. When the matched degradation speed was implemented, the complete degradation of the scaffold happened in the same time span of osteochondral defect repair, i.e. 50 days (Tortorici et al., 2021). The fast and slow speeds were chosen to result in complete scaffold degradation in half (25 days) and double (100 days) the time, respectively, compared to the matched speed. The degradation rates of the “artificial” scaffold were calculated to fulfil the imposed degradation times.

Experimentally, the bulk degradation of several polymers, such as PCL, PDLLA, and PLGA, has been shown to follow a non-linear behavior (Pitt C et al., 1981; Pitt G et al., 1981; Wu & Ding, 2004). Therefore, a simulation of scaffold bulk degradation by hydrolysis that was closer to the *in vivo* or *in vitro* cases was subsequently implemented based on the experimental data reported in literature.

#### Hydrolytic Degradation by Surface Erosion

Experimentally, a hydrolytic degradation process by surface erosion results in a thinning of the scaffold features without alterations of the bulk properties (Middleton & Tipton, 2000). Here, surface erosion was modelled by reducing the amount of scaffold material without changes in the scaffold material properties. Each element could be occupied by a mixture of tissues and scaffold material, where the parameter quantifying the amount of each species was the corresponding volume fraction. Thus, surface erosion was simulated by a linear decrease in the volume fraction of the scaffold material (expressed as percentage of the total volume of an individual element), as indicated by Equation 3:

$$N_{t}=N_{0}-k_{N}t$$ (3)

Where *N*_*t*_ and *N*_*0*_ (= 50% due to the porosity of the scaffold material) are the volume fraction of the scaffold at a time *t* and at the beginning, respectively, and *k*_*N*_ is the degradation rate, whose values are reported in Table 4. Moreover, the reduction in volume fraction was applied only to the elements situated on the scaffold surface (Figure 2D left). When the volume fraction of the scaffold in an element was zero (completely degraded), the elastic modulus of the scaffold material in that element was set to zero (Figure 2B left).

**TABLE 4:** Degradation rates of volume fraction (*k_N_*) and elastic modulus (*k_E_*) of scaffold material for the three degradation modalities and the three degradation speeds investigated with the “artificial” scaffold. The time of complete scaffold degradation for each degradation speed is indicated in brackets. The volume fraction is expressed as percentage of the volume of one element.

Degradation modality	Fast (25 days)	Matched (50 days)	Slow (100 days)
Surface erosion	Surface elements: *k*_*N*_ = 4%/day	Surface elements: *k*_*N*_ = 2%/day	Surface elements: *k*_*N*_ = 1%/day
Bulk degradation	All elements	All elements	All elements
	• *k*_*E*_ = 40 MPa/day	• *k*_*E*_ = 20 MPa/day	• *k*_*E*_ = 10 MPa/day
	• *k*_*N*_ = 20%/day	• *k*_*N*_ = 10%/day	• *k*_*N*_ = 5%/day
Bulk degradation with autocatalysis	Surface elements	Surface elements	Surface elements
	• *k*_*E*_ = 40 MPa/day	• *k*_*E*_ = 20 MPa/day	• *k*_*E*_ = 10 MPa/day
	• *k*_*N*_ = 20%/day	• *k*_*N*_ = 10%/day	• *k*_*N*_ = 5%/day
	Bulk elements	Bulk elements	Bulk elements
	• *2k*_*E*_ = 80 MPa/day	• *2k*_*E*_ = 40 MPa/day	• *2k*_*E*_ = 20 MPa/day
	• *k*_*N*_ = 40%/day	• *k*_*N*_ = 20%/day	• *k*_*N*_ = 10%/day

#### Hydrolytic Degradation by Bulk Degradation

In a scaffold undergoing bulk degradation, the molecular weight is homogeneously reduced without alterations in volume (Casalini, 2017). Moreover, the mechanical properties of a polymer are closely related to its molecular weight (Nunes et al., 1982). Here, direct proportionality was assumed between molecular weight and the compressive elastic modulus of the scaffold material, as indicated by Equation 4:

$$E_{t}\propto Mw_{t}$$ (4)

Where *E*_*t*_ and *Mw*_*t*_ are the elastic modulus and the molecular weight, respectively, of the scaffold material at a time *t*. Thus, bulk degradation was simulated by a linear and homogeneous reduction of the elastic modulus of the scaffold material (Figure 2B middle), following Equation 5:

$$E_{t}=E_{0}-k_{E}t$$ (5)

Where *E*_*t*_ and *E*_*0*_ (= 1,000 MPa) are the elastic modulus of the scaffold material at a time *t* and at the initial time, respectively, and *k*_*E*_ is the degradation rate (Table 4).

Eventually, also scaffolds undergoing bulk degradation begin losing mass and volume, i.e., begin eroding (see definition in Table 1). For example, the onset of mass loss for PCL has been observed at a molecular weight of 5,000 g/mol (Pitt C et al., 1981). This result was reported to be independent of the initial molecular weight. As the degradation behavior of PCL with an initial molecular weight of approximately 50,000 g/mol was described in detail (Pitt C et al., 1981), a threshold of 10% of the initial molecular weight was here taken as reference for a simplified model of the onset of erosion. Taking into account the direct proportionality between elastic modulus and molecular weight assumed here (see Equation 4), the loss of scaffold volume fraction in the model of bulk degradation begun when *E*_*t*_ was 10% of the initial material elastic modulus. In this case, the erosion process was modelled by applying Equation 3 to all elements of the scaffold, independently of their position on the surface or in the bulk of the struts, with a degradation rate *k*_*N*_ enabling the complete degradation of the scaffold in the imposed time (Table 4). When the volume fraction of the scaffold in an element was completely degraded, the elastic modulus of the scaffold material in that element was set to zero.

#### Hydrolytic Degradation by Bulk Degradation With Autocatalysis

Autocatalysis is established when the acidic degradation by-products accumulate in the bulk of the polymeric device, fostering a faster reduction of the molecular weight in the inner regions compared to the surface (Middleton & Tipton, 2000). Taking into account the direct proportionality between molecular weight and elastic modulus assumed here (see Equation 4), the establishment of autocatalysis was modelled by imposing a greater reduction in elastic modulus to the elements in the bulk of the scaffold struts compared to the elements on the surface. It has been reported that after 2 weeks of *in vivo* degradation, the molecular weight on the surface of PDLLA devices was almost two times higher than the one in the bulk (Therin et al., 1992). Therefore, the reduction in elastic modulus of the elements on the surface of the scaffold struts were calculated with Equation 5, while Equation 6 was applied to the elements in the bulk of the struts:

$$E_{t}=E_{0}-2k_{E}t$$ (6)

Also in this case, a reduction in volume fraction of the scaffold material begun when the elastic modulus reached 10% of its initial value (Figures 2B,D right), as described above for bulk degradation without autocatalysis. The degradation rates of volume fraction and elastic modulus of the scaffold material when autocatalysis was implemented are listed in Table 4. Moreover, the elastic modulus of the scaffold material was set to zero in the elements with complete volume fraction degradation.

#### Hydrolytic Degradation Based on Experimental Observations

After an initial evaluation with simplified degradation behaviors, the model of scaffold degradation was employed to more closely reproduce experimentally observed degradation phenomena. The reduction in polymeric molecular weight during bulk degradation without autocatalysis has been reported to follow an exponential law in several cases: for example, in porous scaffolds produced from PDLLA and PLGA (Wu & Ding, 2004) and in film and capsules of PCL (Pitt C et al., 1981) and PDLLA (Pitt G et al., 1981). Based on the assumption of direct proportionality between molecular weight and elastic modulus applied here (see Equation 4), this experimental observation was implemented in the model of scaffold degradation with Equation 7:

$$E_{t}=E_{0}e^{-k_{e}t}$$ (7)

Where *k*_*e*_ is the exponential degradation rate. Equation 7 was applied to the “artificial” scaffold with strut-like architecture previously investigated with the linear degradation laws described in the sections above. Moreover, Equation 7 was used to simulate bulk degradation without autocatalysis, and was thereby applied to all scaffold elements independently of their position on the surface or in the bulk of the struts.

PCL films and capsules were reported to have an exponential degradation rate of 0.003 days^−1^ *in vivo* (Pitt C et al., 1981). No autocatalysis was observed in PCL devices *in vivo* (Pitt C et al., 1981; Lam et al., 2009). Moreover, the degradation of PCL does not depend on the surface area, as shown by comparing the *in vitro* degradation of PCL films and micro-particles (Chen et al., 2000). Therefore, the experimentally measured value of the PCL degradation rate was considered appropriate to describe the exponential degradation of the scaffold modelled here. Thus, a value of 0.003 MPa/day was assigned to a slow exponential degradation rate (*k*_*e,slow*_).

The degradation rate of PDLLA films and capsules *in vivo* was measured to be 0.00841 days^−1^ (Pitt G et al., 1981). However, PDLLA has been observed to undergo autocatalysis (Therin et al., 1992). Therefore, the size of a device produced from PDLLA will influence its degradation rate (Grizzi et al., 1995). Porous PDLLA scaffolds (porosity >80%) were reported to have an *in vitro* exponential degradation rate of 0.023 weeks^−1^, corresponding to 0.003 days^−1^, without the establishment of autocatalysis (Wu & Ding, 2004). Therefore, the exponential degradation rate *k*_*e,slow*_ with a value of 0.003 MPa/day was deemed appropriate to model the degradation of a porous scaffold produced from both PCL and PDLLA.

The *in vitro* exponential degradation rate of porous PLGA scaffolds (lactic/glycolic acid molar ratio of 75/25) was measured to be 0.153 weeks^−1^, corresponding to 0.022 days^−1^, without autocatalysis phenomena (Wu & Ding, 2004). Therefore, a fast exponential degradation rate (*k*_*e,fast*_) with value of 0.022 MPa/day was implemented to simulate the degradation of porous scaffolds produced from PLGA.

With the *k*_*e,slow*_ exponential degradation rate, the onset of erosion at 10% of the initial elastic modulus value was calculated to take place after more than 2 years of degradation (specifically, at day 768). To enable a comparison with the simplified degradation behaviors studied here while keeping the evaluation within reasonable computational times, the model of PCL and PDLLA bulk degradation with exponential degradation law was evaluated until day 125. When the *k*_*e,fast*_ exponential degradation rate was implemented, the onset of erosion was calculated to take place at day 105. Given the similar times of complete degradation between the simulated exponential degradation of PLGA and the simplified slow bulk degradation, the same erosion rate (*k*_*N*_ = 5%/day) was implemented to model the erosion of the PLGA porous scaffolds. The set up of the models implementing the experimentally-derived exponential bulk degradation is summarized in Table 5.

**TABLE 5:** Parameters of the models implementing the exponential bulk degradation. PCL: poly (ε-caprolactone); PDLLA: poly (D,L-lactic acid); PLGA: poly (lactide-co-glycolide); *k_e_*: exponential degradation rate; *k_N_*: erosion rate (as percentage of the total volume fraction of one element/day).

Modelled material	Degradation rate	Time of complete scaffold degradation
Porous PCL or PDLLA scaffolds	• *k*_*e,slow*_ = 0.003 MPa/day	>2 years
Porous PLGA scaffold	• *k*_*e,fast*_ = 0.022 MPa/day	Day 115
	• *k*_*N*_ = 5%/day	

### Evaluation of Computational Result

In the presented model, tissue formation was determined by cellular number and distribution, while the distribution of *S* (mechanical stimulus) indicated the tissues whose formation or resorption would have been favored by the mechanical environment at a specific day. Due to the applied mechano-biological rules of tissue formation, cellular distributions were not independent from the distribution of *S*, but they might differ from it before reaching the equilibrium state, i.e. the full repair of the defect. Therefore, results were evaluated by comparing the distribution of MSCs, osteoblasts, chondrocytes, and fibroblasts to derive the distribution of granulation tissue, bone, cartilage, and fibrous tissue, respectively. However, it was previously observed that cellular distributions at the equilibrium state matched the prediction of tissue formation based on *S* (Tortorici et al., 2021). Thus, the predicted repair outcome was here visualized by the distribution of *S* at the end of the repair process.

Some of the modelled degradation cases never reached the equilibrium state due to non-convergence of the finite element analysis at some day of the iterative repair process. The non-convergence of the model was caused by the excessive deformation of elements belonging to the scaffold during its degradation. This simulation outcome was considered equivalent to a mechanical failure of the scaffold *in vivo* and was not further analyzed.

The stiffness of the scaffold during the degradation process was determined by a separate FE analysis, which modelled the scaffold alone in its three-dimensional form (i.e. three concentric rings, Supplementary Figure S1). The overall elastic modulus of the scaffold material (*E*_*Scaffold*_) was calculated taking into account both the elastic modulus (*E*_*t*_) and volume fraction (*N*_*t*_) at a given time, as indicated by Equation 8:

$$E_{Scaffold}=E_{t}*N_{t}$$ (8)

Therefore, different *E*_*Scaffold*_ were assigned to elements on the surface and in the bulk of the struts when modelling non-homogeneous degradation processes, e.g. surface erosion. A 3% compressive displacement (*Δx*) was applied and the resulting reaction force (*RF*) was measured, enabling the calculation of the scaffold stiffness (*K*) with Equation 9:

$$K=\frac{RF}{\Delta x}$$ (9)

## Results

The influence of polymeric scaffold degradation on the mechanics-dependent repair of osteochondral defects was investigated *in silico* by simulating three modalities of hydrolytic degradation: surface erosion, bulk degradation, and bulk degradation with autocatalysis. For each degradation modality, three speeds were investigated: fast, matched, and slow, causing the complete degradation of the scaffold in 25, 50, and 100 days, respectively. The repair outcome was evaluated at day 125. Moreover, bulk degradation with an exponential loss of mechanical competence was implemented, simulating the experimentally-observed degradation rates of porous scaffolds produced from PCL or PDLLA (which were reported to have comparable exponential degradation rates) and from PLGA. The following sections report the results obtained for each degradation modality and each degradation speed.

### Degradation by Surface Erosion

Scaffold degradation by surface erosion was modelled by reducing the volume fraction occupied by the scaffold material in the elements situated on the scaffold surface. The distribution of octahedral shear strain (γ) and the prediction of tissue formation over the repair process prior to equilibrium are reported in Supplementary Figure S2.

When the fast degradation by surface erosion was implemented, the model predicted the mechanical failure of the scaffold at day 24, i.e. one day before its complete degradation (Table 6). Therefore, the results of this simulation were not further analyzed.

**TABLE 6:** Models for which the mechanical failure of the scaffold prior to complete defect repair was predicted.

Degradation modality	Degradation speed	Day of complete scaffold degradation	Model-specific events	Day of mechanical failure
Surface erosion	Fast	25	• Complete degradation of first layer of surface elements: day 13	24
Bulk degradation with autocatalysis	Fast	25	• Complete degradation of elements in strut bulk: day 13	14
			• Onset of erosion: oSurface: day 23	
			oBulk: day 12	
	Matched	50	• Complete degradation of elements in strut bulk: day 25	26
			• Onset of erosion: oSurface: day 45	
			oBulk: day 23	
	Slow	100	• Complete degradation of elements in strut bulk: day 50	67
			• Onset of erosion: oSurface: day 90	
			oBulk: day 45	

**FIGURE 3:**
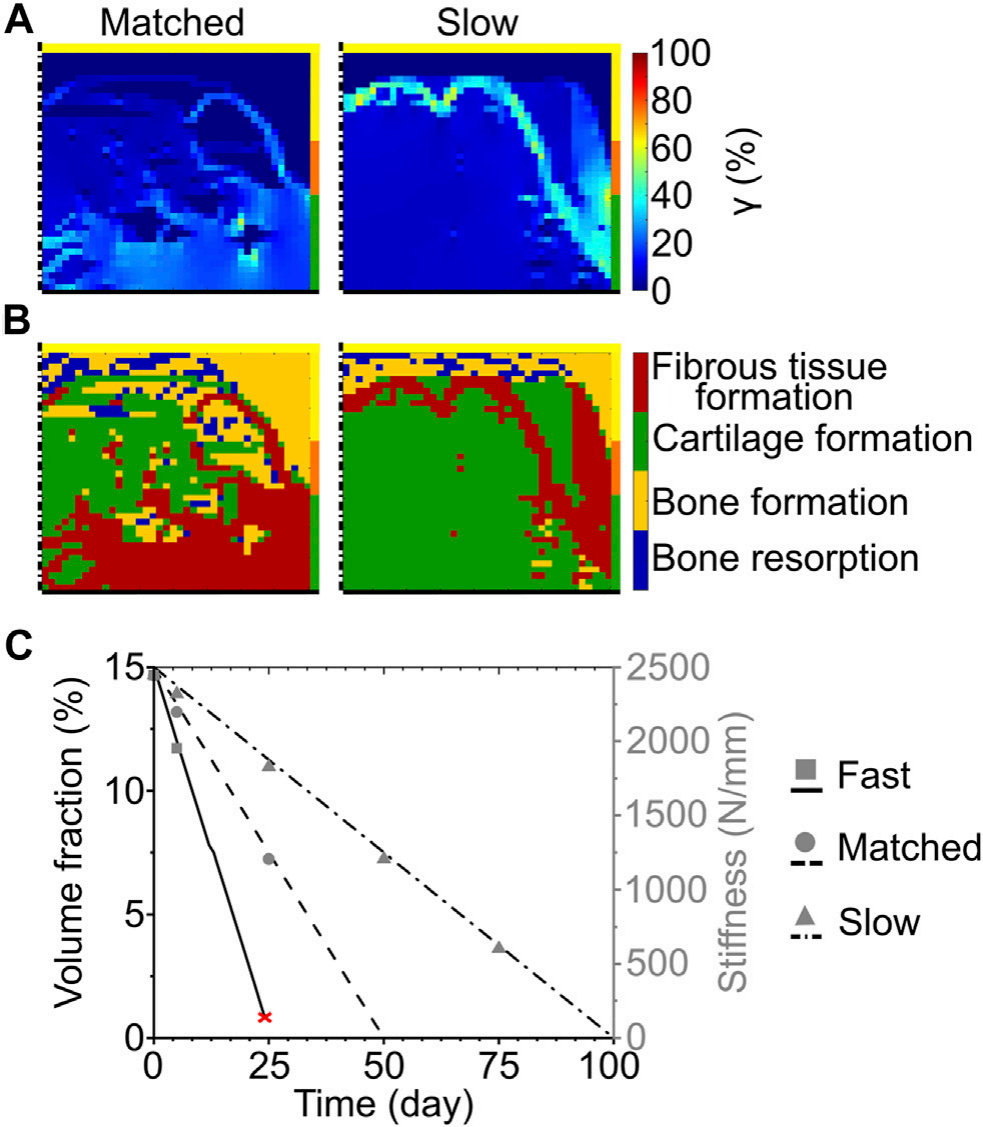
Influence of scaffold degradation by surface erosion on osteochondral defect repair. **(A)** Distribution of octahedral shear strain (*γ*); **(B)** prediction of tissue formation based on the mechanical stimulus (*S*). The left and right columns show the repair outcome with matched and slow degradation speed at day 125. The yellow, orange, and green borders indicate the neighboring healthy tissues of cancellous bone, subchondral bone, and cartilage, respectively. The dash-dot and solid black lines highlight the axis of symmetry and the articular surface, respectively; **(C)** Volume fraction (as percentage of the total defect volume) and stiffness of the scaffold during degradation with different speeds. The red cross marks the predicted mechanical failure of the scaffold. Lines and symbols refer to volume fraction and stiffness, respectively.

At the completion of the repair process, the scaffold degrading by surface erosion with matched degradation speed resulted in very low (<1%) mechanical strains (γ) at the proximal base and side of the defect (Figure 3A left). Intermediate (≈5–8%) and high (≈10–30%) values of strain were predicted in the middle region and at the articular surface, respectively (Figure 3A left). The predicted repair outcome resulted in fibrous tissue formation at the articular surface, cartilage formation in the central region, and bone formation at the side and base of the defect (Figure 3B left). The interfaces between the different formed tissues were not clear-cut, showing ample areas of mixed tissue growth and traces of bone resorption at the base of the defect.

The implementation of the slow degradation speed resulted in very low mechanical strains (γ < 1%) at the base and side, while the rest of the defect experienced intermediate strains (γ ≈ 7%) (Figure 3A right). The regions of low and intermediate strains were separated by an area subjected to high values of γ, ranging from 10 to 64% (Figure 3A right). The corresponding mechanics-dependent prediction of the repair outcome was the formation of cartilage in most of the defect, with bone growing at the proximal base and side and an intermediate region of fibrous tissue formation (Figure 3B right).

The volume fraction of the scaffold showed a linear decrease for each investigated speed, as imposed by the applied degradation model (Figure 3C, black lines). Concomitantly, the stiffness of the scaffold decreased from the initial value of 2445 N/mm (Figure 3C, gray symbols). Although at early time points the stiffness of the scaffold was comparable amongst the different degradation speeds (e.g., 1951 N/mm for fast, 2197 N/mm for matched, and 2320 N/mm for slow degradation speeds at day 5), it dropped rapidly to 0 N/mm when the fast and matched degradation speeds were imposed. With the slow degradation speed, the scaffold stiffness maintained high values (>500 N/mm) until a late time point of the repair process (day 75).

### Degradation by Bulk Degradation

The bulk degradation of the scaffold was modelled by a homogenous reduction in the elastic modulus of the scaffold material. When the material elastic modulus reached 10% of its initial value, it determined the onset of scaffold erosion, leading to a reduction in the volume fraction of the scaffold material until its complete disappearance. The distribution of mechanical strain (γ) and the prediction of tissue formation based on the mechanical stimulus prior to equilibrium are reported in Supplementary Figure S3.

**FIGURE 4:**
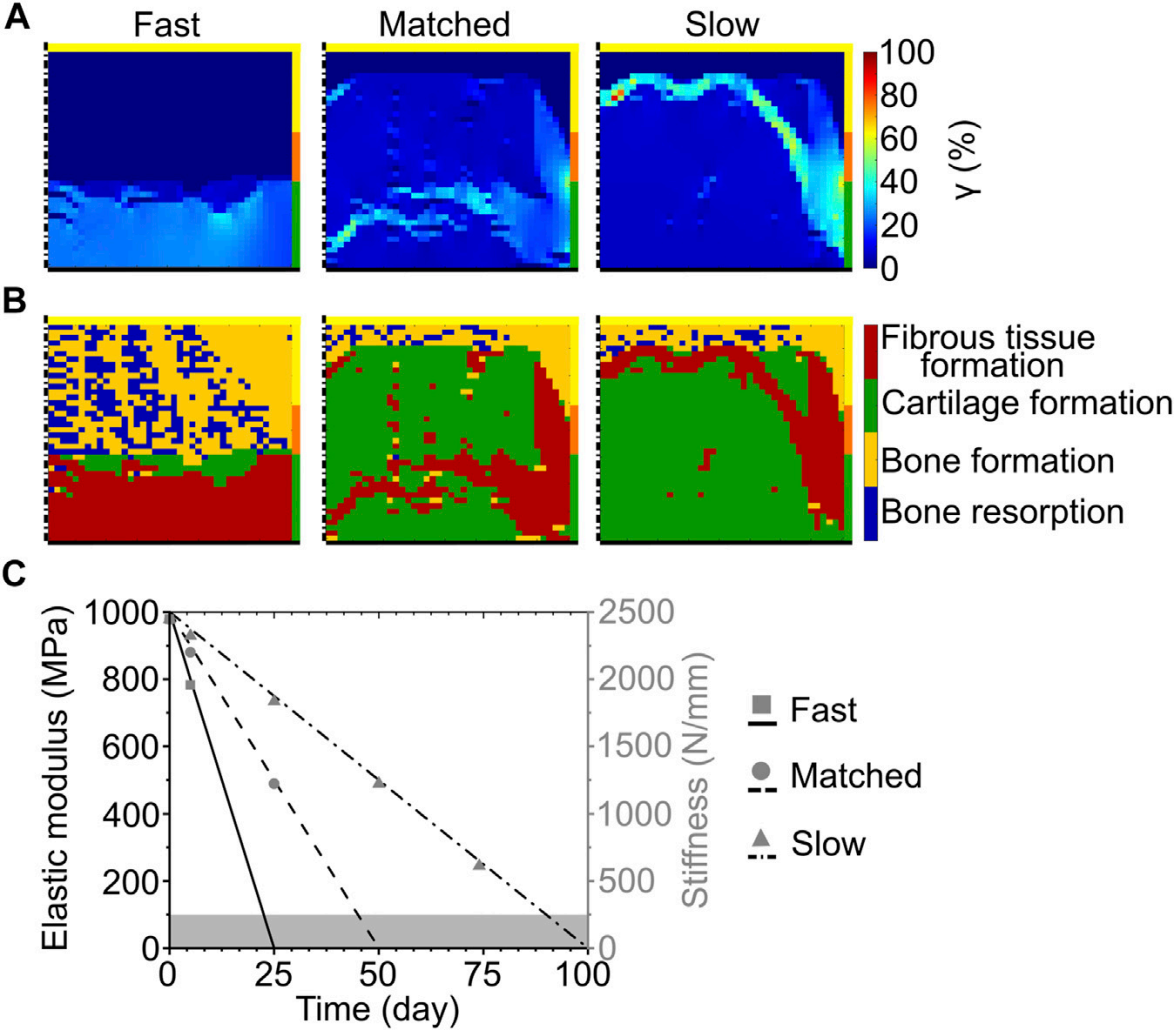
Influence of scaffold degradation by bulk degradation on osteochondral defect repair. **(A)** Distribution of octahedral shear strain (*γ*); **(B)** prediction of tissue formation based on the mechanical stimulus (*S*). The left, middle, and right columns show the repair outcome with fast, matched and slow degradation speed, respectively, at day 125. The yellow, orange, and green borders indicate the neighboring healthy tissues of cancellous bone, subchondral bone, and cartilage, respectively. The dash-dot and solid black lines highlight the axis of symmetry and the articular surface, respectively; **(C)** Material elastic modulus and scaffold stiffness during degradation with different degradation speeds. The grey area marks the region of concomitant scaffold erosion. Lines and symbols refer to elastic modulus and stiffness, respectively.

At the completion of the repair process, the scaffold with fast bulk degradation resulted in very low (<1%) values of γ in most of the defect (Figure 4A left). At the articular surface, however, γ ranged from 20 to 35% (Figure 4A left). The corresponding repair outcome was the formation of fibrous tissue at the articular surface, the growth of bone at the side, and the establishment of a region of mixed bone resorption and bone apposition in the middle and at the proximal base of the defect (Figure 4B left). Cartilage formation was predicted only in a small central area (Figure 4B left).

When the matched bulk degradation was implemented, γ assumed very low (<1%) values at the proximal base and side, while most of the defect experienced intermediate strain values of approximately 4–8% (Figure 4A middle). High values of γ (≈18–40%, with local peaks up to 52%) were found in a narrow band crossing the distal half of the defect and at the interface with healthy subchondral bone and cartilage (Figure 4A middle). This distribution of γ resulted in the prediction of cartilage formation in the defect, with bone apposition at the base and side and fibrous tissue formation in the region of high strain (Figure 4B middle).

Similarly, the values of γ were very low (<1%) at the base and side and intermediate (≈7%) in most of the defect with slow bulk degradation (Figure 4A right). A band of high γ (20–40%, with local peaks up to 90%) was observed in this case in the proximal half of the defect and at the interface with the surrounding healthy tissues (Figure 4A right). The corresponding repair prediction was the formation of cartilage in most of the defect, with bone apposition and fibrous tissue formation in the regions of very low and high γ, respectively (Figure 4B right).

In accordance with the imposed degradation model, the elastic modulus of the scaffold material linearly decreased throughout the repair process (Figure 4C, black lines). Interestingly, despite the different modality by which the mechanical competence of the scaffold was lost, the stiffness of the scaffold throughout the bulk degradation process (Figure 4C, grey symbols) matched that observed during the surface erosion process (Figure 3C, grey symbols).

### Degradation by Bulk Degradation With Autocatalysis

Autocatalysis was modelled by a faster reduction in material elastic modulus in the bulk of the scaffold struts compared to the surface. Also in this case, scaffold erosion begun when the elastic modulus of the scaffold material reached 10% of its initial value. The distribution of γ and the prediction of tissue formation based on the mechanical stimulus prior to equilibrium are reported in Supplementary Figure S4.

None of the models implementing autocatalysis reached the equilibrium state, i.e. the full repair of the osteochondral defect, independently of the imposed degradation speed (Table 6). Typically, the mechanical failure occurred the day after the complete degradation of the scaffold bulk, when the struts of the scaffold became an “empty shell”. The model with slow degradation was an exception, with the mechanical failure happening at day 67, i.e. 17 days after the complete degradation of the bulk of the struts.

### Degradation Based on Experimental Data

The hydrolytic bulk degradation of porous scaffolds produced from several synthetic polymers, such as PCL, PDLLA, and PLGA, has been reported to follow an exponential law. Therefore, scaffold degradation by bulk degradation was modelled by implementing an exponential reduction in elastic modulus with experimentally-derived degradation rates. The experimentally-derived degradation rates simulated the degradation of porous scaffold produced from PCL or PDLLA, denoted as slow, and from PLGA, denoted as fast. The distribution of γ and the prediction of tissue formation based on the mechanical stimulus prior to equilibrium are reported in Supplementary Figure S5.

**FIGURE 5:**
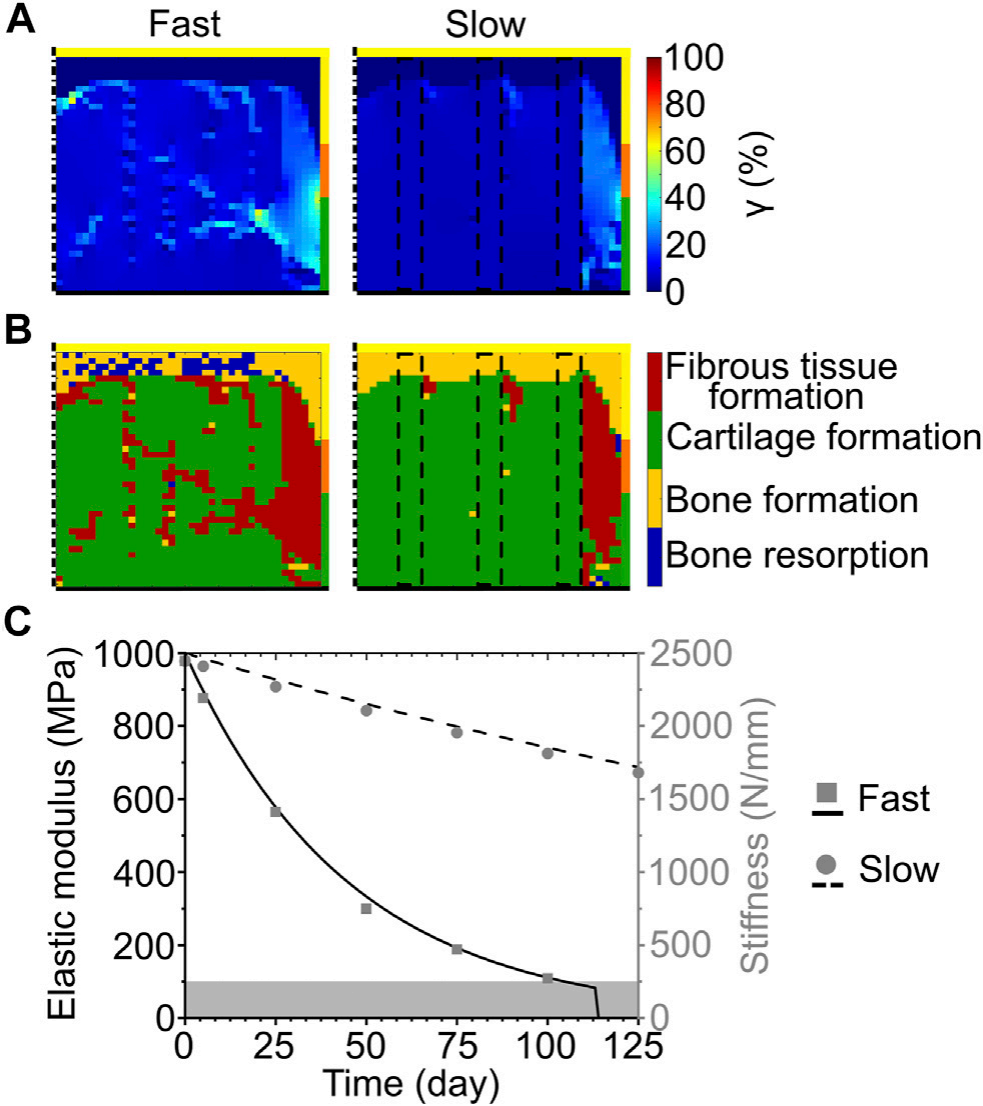
Influence of scaffold degradation with exponential bulk degradation on osteochondral defect repair. **(A)** Distribution of octahedral shear strain (*γ*); **(B)** prediction of tissue formation based on the mechanical stimulus (*S*). All images refer to day 125. The right column shows the outcome obtained with the exponential degradation rate of porous PCL and PDLLA scaffolds (slow), while the left column refers to porous PLGA scaffolds (fast). The yellow, orange, and green borders indicate the neighboring healthy tissues of cancellous bone, subchondral bone, and cartilage, respectively. The dash-dot and solid black lines highlight the axis of symmetry and the articular surface, respectively. The black dashed lines mark the scaffold struts; **(C)** Material elastic modulus and scaffold stiffness during degradation with the fast and slow exponential degradation rate. The grey area marks the region of concomitant erosion. Lines and symbols refer to elastic modulus and stiffness, respectively.

When the slow exponential degradation rate was implemented, the complete degradation of the scaffold material was predicted after more than 2 years. The repair process was studied only until day 125 to enable the comparison with the simplified degradation cases. At this time point, γ showed very low values (<1%) at the base of the defect, intermediate values (≈4–7%) in the central region and at the articular surface, and high values (≈20–30% with a peak of 38%) at the interface with healthy tissues (Figure 5A right). The prediction of tissue formation based on S was the growth of cartilage in the defect, with a thin layer of subchondral bone at the base and fibrous tissue at the interface with the healthy tissues (Figure 5B right). At day 125, the scaffold maintained a high material elastic modulus of 687 MPa (Figure 5C, black dashed line) and its volume fraction was 15% of the total defect volume. Moreover, the scaffold maintained a high stiffness of 1680 N/mm (Figure 5C, gray circles). Although the scaffold was not fully degraded at day 125, tissue formation reached an equilibrium already at approximately day 75. In fact, a maximum difference of 5% was found between the amounts of tissues formed at day 75 and at day 125.

Implementing the fast exponential degradation rate yielded similar results, although with slightly increased values of γ. In fact, γ assumed very low (<1%), intermediate (≈7–10%), and high (≈20–40%, with local peaks up to 60%) values at the base, middle, and side of the defect, respectively (Figure 5A left). As a consequence of the higher values of γ, a greater amount of fibrous tissue was predicted to form in the defect implanted with the scaffold degrading like PLGA (fast exponential degradation, Figure 5B left) compared to the one degrading like PCL or PDLLA (slow exponential degradation, Figure 5B right). Nonetheless, the repair outcome in the two cases was similar, with cartilage forming in the defect and bone growth at the base, although with small regions of bone resorption (Figure 5B left). The elastic modulus of the scaffold material, in accordance with the imposed degradation model, decreased exponentially until day 114, when the completion of the erosion process caused the scaffold material to completely disappear (Figure 5C, black solid line). Concomitantly, the stiffness of the scaffold decreased, reaching a value of 271 N/mm at day 100 (Figure 5C, gray squares), shortly prior to the onset of erosion at day 105.

## Discussion

Scaffold-based tissue engineering strategies aim at supporting the healing of osteochondral defects by overcoming the limitations of current clinical treatments (Davis et al., 2021). However, an identification of the ideal mechanical and architectural features of a scaffold for osteochondral defect healing is still lacking. Promising mechanical and architectural properties of a scaffold for osteochondral defect healing were previously identified *in silico* by employing an axisymmetric model of a knee femoral condyle, in which tissue formation was determined by a mechanical stimulus computed from octahedral shear strain and fluid velocity (Tortorici et al., 2021). However, this model did not provide any insights concerning the influence of scaffold degradation, which has the potential to alter both the mechanical and the architectural characteristics of a device. In this study, the cited model was further developed to simulate the degradation of a synthetic polymeric scaffold with strut-like architecture by three modalities (surface erosion, bulk degradation, and bulk degradation with autocatalysis) and with three different degradation speeds (faster, equal or slower than the tissue repair process). The *in silico* evaluations suggested that, in the absence of autocatalysis, the speed of scaffold degradation, rather than its modality, is the factor having a predominant influence on the mechanics-dependent tissue formation. Specifically, times of full scaffold degradation longer than the expected time of completion of the tissue repair process were found to foster the best repair outcomes in the investigated model.

Initially, the three different modalities of polymeric scaffold degradation were compared to assess whether one of them presented particular advantages or disadvantages for the repair of osteochondral defects. Specifically, simplified algorithms with linear reductions in either volume fraction or elastic modulus for surface erosion and bulk degradation (with or without autocatalysis), respectively, were investigated. For bulk degradation and bulk degradation with autocatalysis, the scaffold begun the erosion process (leading to the complete disappearance of the device) when its material elastic modulus reached 10% of the initial value. Thus, the simplified algorithms reproduced the experimental observations that surface erosion causes thinning of the scaffold features without alterations of molecular weight (here considered directly proportional to the material elastic modulus, see Equation 4) and that bulk degradation results in a homogeneous reduction in molecular weight, and thereby of elastic modulus according to Equation 4, without alterations in the volume of the device up to an advanced state of degradation (Middleton & Tipton, 2000; Casalini, 2017). When autocatalysis was modelled, the elements in the bulk of the scaffold struts had a faster reduction in molecular weight, i.e. in elastic modulus, than those on the surface, also in this case reproducing experimental observations (Therin et al., 1992). Therefore, surface erosion and bulk degradation resulted in a transfer of the load-bearing function from the scaffold to the newly formed tissues in the model by two different mechanisms. In surface erosion, the biological tissues could gradually grow in areas previously occupied by the scaffold, whose load-bearing ability was reduced by the physical disappearance of the scaffold material. In bulk degradation, the amount of scaffold material remained unaltered for the majority of the degradation process and the transfer of the load-bearing function to the biological tissues happened by a softening of the scaffold material itself.

Previous analyses performed with the same computational model studied the mechanics-dependent repair process of an untreated osteochondral defect and of an osteochondral defect implanted with a non-degradable scaffold, which had the same mechanical and morphological properties of the scaffold investigated here (Tortorici et al., 2021). In the mentioned study, the repair of untreated osteochondral defects was predicted to develop fibrous tissue at the articular surface, with bone resorption mixed with bone formation in the middle and proximal regions, and lateral bone apposition. The non-degradable scaffold fostered an improved repair, as cartilage formed in most of the defect (and specifically at the articular surface) and a thin subchondral bone layer was established throughout the proximal base, although fibrous tissue was predicted to form at the interface with the surrounding healthy tissues. The improved repair outcome fostered by the scaffold was ascribed to its mechanical support, which reduced the strain in the defect, and to its architecture, which enabled load transmission to the defect base.

When a fast bulk degradation of the scaffold was implemented, the repair outcome was analogous to the one predicted for the untreated osteochondral defect (Figure 4B left), indicating that the mechanical support provided by the scaffold lasted a time too short to significantly steer tissue formation. On the other hand, both matched and slow bulk degradation resulted in a repair outcome similar to the non-degradable scaffold, although with higher amounts of fibrous tissue (Figure 4B middle and right, respectively). The increased formation of fibrous tissue could be ascribed to higher strains generated by the progressive decrease in stiffness of the scaffold (Figure 4C). Interestingly, the mechanical failure of the scaffold was never predicted when bulk degradation was implemented, independently of the degradation speed. This result was ascribed to the gradual and homogenous change in mechanical properties of the scaffold, which avoided the excessive straining of individual scaffold elements.

When the scaffold degraded by surface erosion, the fast degradation speed was predicted to induce mechanical failure of the scaffold (Table 6). As the failure occurred 1 day prior to complete scaffold degradation, it could be ascribed to the low thickness of the struts and their extremely increased porosity, which derived from the reduction in the volume fraction occupied by the scaffold material (Figure 3C). Mechanical failures were observed neither with the matched nor with the slow surface erosion. The repair outcome with the matched surface erosion was intermediate between the ones previously obtained in the untreated defect and with the non-degradable scaffold (Tortorici et al., 2021). In fact, a continuous subchondral bone layer formed and cartilage was found in the middle of the defect (Figure 3B left); however, the articular surface was occupied by fibrous tissue. Thus, the scaffold undergoing matched surface erosion did not provide sufficient mechanical support to the defect to avoid major fibrous tissue formation. Interestingly, the repair outcome with slow surface erosion was analogous to the one with slow bulk degradation (compare Figure 3B right to Figure 4B right). Although the overall stiffness of surface-eroding and bulk-degrading scaffolds was comparable between all imposed degradation speeds throughout the simulation (compare Figure 3C and Figure 4C), a similar repair outcome was achieved only with the implementation of the slow degradation speed. This result seems to indicate that the modality of degradation (either surface erosion or bulk degradation without autocatalysis) for scaffolds with strut-like architecture does not have an influence on the mechanics-dependent repair process, as long as the scaffold provides mechanical support for a sufficiently long time. Notably, such a sufficiently long time was identified in this computational model not as the time of expected defect healing, i.e. 50 days, but as long as 100 days. The similarity of overall scaffold stiffness during bulk degradation and surface erosion observed in this model was ascribed to the low thickness of the strut-like architecture, where the strut width was composed of 50% surface and 50% bulk elements. Thus, the reduction in scaffold material during surface erosion had a comparable influence on the overall mechanical properties to a homogenous reduction in material elastic modulus. This result is not expected to hold true for bulkier devices, for which surface erosion might have a reduced influence on the overall mechanical properties. It is important to notice that when both surface erosion and bulk degradation (without autocatalysis) with slow degradation speed were implemented, a layer of fibrous tissue developed at the interface between soft and hard tissues, probably deriving from increased strain values in this area resulting from the progressive loss of mechanical support from the scaffold. To avoid this effect, the architecture of the scaffold could be modified to one that provides a higher resistance against deformation in the early phases of degradation: for example, a grid-like architecture that reduces displacements both in the direction of the applied load and perpendicular to it has been previously indicated as favorable (Tortorici et al., 2021).

The results of the computational model clearly indicated that the instauration of autocatalysis is critical, as the mechanical failure of the scaffold with strut-like architecture was consistently predicted prior to full repair of the osteochondral defect, independently of the imposed degradation speed (Table 6). Such a markedly negative effect depended also on the architecture of the scaffold, which was composed only of thin (0.5 mm thickness) vertical struts (Figure 1A). As the bulk of the scaffold struts was completely degraded, the two surface layers of each strut effectively became independent pillars with the same height of the original strut (5 mm), but a much reduced thickness of 0.125 mm, corresponding to the width of one element in the mesh of the finite element model. This alteration of the height-to-thickness ratio, combined with the progressive decrease in material elastic modulus, made the scaffold struts more susceptible to buckling phenomena or to breakages due to excessive deformation. In particular, buckling strongly depends on the aspect ratio of the loaded body and may cause failures even if the yield stress of the material has not been exceeded (Beer et al., 2015). Therefore, scaffold architectures different than the one investigated here might better sustain loads even in presence of autocatalysis, for example if featuring connecting elements between vertical struts and/or having oblique rather than vertical struts. Nonetheless, the transformation of the scaffold into a structure consisting only of a thin outer layer, and the deriving reduction in mechanical competence, could be expected when employing materials that undergo bulk degradation with autocatalysis. The precise thickness of the remaining outer layer depends on several factors, e.g. the diffusion speed of the involved molecules or oligomers and the cleavage rate of the chemical bonds (Cameron & Kamvari-Moghaddam, 2008).

Bulk degradation without autocatalysis was also modelled as an exponential decrease in molecular weight (and thereby in elastic modulus, see Equation 4) based on the available literature data. This analysis enabled an evaluation of the influence of degradation that came closer to an experimental case. Specifically, the degradation behavior of porous scaffolds made from PCL or PDLLA and from PLGA was implemented. When the PCL- or PDLLA-like degradation was studied (slow exponential degradation rate), the complete disappearance of the scaffold was calculated to happen after more than 2 years. Therefore, the scaffold material was still present at the last investigated time point (day 125). However, tissue formation had already reached equilibrium at day 75, meaning that variations in tissue distributions from the results here reported might be expected only after the onset of erosion. As the scaffold material maintained a high elastic modulus (>600 MPa, Figure 5C) throughout the investigated time, the mechanics-dependent repair of the osteochondral defect (Figure 5B right) was analogous to the one obtained with the non-degradable scaffold (Tortorici et al., 2021), which was regarded as a positive outcome. It remains to be ascertained whether the onset of erosion would drastically alter this result. However, in the *in vivo* case, the production of extra-cellular matrix would likely stabilize the tissue configuration that was built during the long time of scaffold degradation. Thus, the repair tissues would gradually become independent of the mechanical support of the scaffold, reducing the risk of large variations in tissue distribution in consequence of the full degradation of the scaffold material.

A similar repair of the osteochondral defect was predicted with the material degrading as a porous PLGA scaffold (fast exponential degradation rate), although more fibrous tissue formation and regions of bone resorption were predicted (Figure 5B left). In this case, the degradation was faster and the scaffold material had completely disappeared at the last investigated time point (day 125, Figure 5C). The results obtained with the experimentally-derived bulk degradation rates confirmed the observation performed with the linear degradation rates, i.e. that long times of scaffold degradation are particularly advantageous to the mechanics-dependent tissue formation.

A verification of the computational results by comparison with the *in vivo* tissue formation in osteochondral defects is challenging. Although the *in vivo* degradation behavior of scaffolds or biomaterials has been often studied in subcutaneous (Lam et al., 2008), subdermal (Pitt C et al., 1981; Pitt G et al., 1981), intramuscular (Lam et al., 2008; Therin et al., 1992) or calvarial (Lam et al., 2008) defects, extensive evaluations of scaffold degradation in osteochondral defects, and thereby of degradation-dependent tissue formation, are, to the best of our knowledge, still lacking. When testing biodegradable or bioresorbable scaffolds for osteochondral defect healing, the presence or absence of scaffold remnants at a specific time point is a frequently reported observation on the *in vivo* degradation behavior of the specimens (Athanasiou et al., 1997; Barron et al., 2015; Holland et al., 2005; Hsieh et al., 2018; Ikeda et al., 2009; Reyes et al., 2012; Schlichting et al., 2008). Although important to assess the time of complete disappearance of the investigated device, this observation is not sufficient to establish a comparison with the results of the here presented model in terms of mechanics-dependent tissue formation. Some studies offer an evaluation of the *in vivo* degradation of scaffolds in osteochondral defects based on previous *in vitro* tests. For example, a two-phase PLGA scaffold implanted in goat failed to support tissue growth in the center of the osteochondral defects, an outcome ascribed to the faster degradation of the scaffold core due to the establishment of autocatalysis, as observed *in vitro* (Athanasiou et al., 1997). However, some authors reported differences in the *in vivo* degradation of the scaffolds compared to the results expected from previous *in vitro* evaluations (Holland et al., 2005), or that expected differences in degradation amongst specimens could not be confirmed *in vivo* by visual inspection only (Wang et al., 2014). In another study, two types of PLGA scaffolds were tested in sheep, with the stiffer scaffold with slower degradation resulting in improved subchondral bone formation (Schlichting et al., 2008). Interestingly, the authors suggested that mechanical failures due to the faster degradation of the softer scaffold may have negatively influenced tissue formation, thereby supporting our *in silico* predictions. However, the differences in the initial stiffness and porosity of the investigated scaffolds prevent a clear comparison with our model in terms of mechanics-dependent tissue growth. Therefore, the results here obtained *in silico* can be considered as an indication, while further experimental assessments are needed to confirm these findings. For example, direct comparisons between *in vivo* and simulated tissue repair have been reported for untreated osteochondral defects (Duda et al., 2005) and scaffold-supported large bone defects (Perier-Metz et al., 2020) and could be in future performed also for osteochondral defects treated with biodegradable architectured scaffolds.

As limitation of the here presented model, the “artificial” scaffold configuration must be mentioned. The scaffold was defined “artificial” because it did not model a specific polymer nor scaffold architecture currently investigated in tissue engineering, but its simplified properties were shown to promote regeneration in a previous computational study (Tortorici et al., 2021). The “artificial” scaffold in each investigated case had the same mechanical and architectural properties in its non-degraded state, but the possibility to degrade by each one of the three investigated modalities in turn. This situation was also “artificial” because, in reality, the degradation modality of a polymeric device is determined by both its chemical and architectural features (von Burkersroda et al., 2002). Thus, polymeric scaffolds degrading by different modalities can hardly be expected to have equal properties prior to the onset of degradation. For example, changes in architecture might be required to obtain the same overall stiffness in scaffolds produced from different materials. Further limitations of this study include the simplified degradation behaviors that were implemented, as well as the evaluation of only mechanics-related phenomena. A first simplification in the implemented degradation behaviors was the clear-cut distinction between surface erosion and bulk degradation, while in reality polymeric degradation might involve a combination of the two modalities (Göpferich, 1996). A second simplification was the direct proportionality between decrease in polymeric molecular weight and decrease in material elastic modulus assumed here. Although the loss of mechanical competence is a degradation-related phenomenon, the kinetics of molecular weight reduction and of elastic modulus decrease might differ (Göpferich, 1996). For example, in highly porous PLGA scaffolds, the first stages of *in vitro* degradation were characterized by a constant or slightly increased elastic modulus, while the molecular weight was already subjected to reductions (Wu & Ding, 2004). Furthermore, the linear algorithms and the imposed degradation speeds employed for the comparison of the three degradation modalities were a third simplification, which has been addressed in the second part of this work. In fact, bulk degradation has been reported to follow an exponential behavior for several polymers, such as PCL, PDLLA, and PLGA (Pitt C et al., 1981; Pitt G et al., 1981; Wu & Ding, 2004). Moreover, the degradation coefficients measured experimentally, e.g. for PCL and PDLLA, would cause a much slower scaffold degradation than the one modelled with the fast, matched, and slow degradation speeds, as shown when the experimentally-derived bulk degradation was implemented (Figure 5C). Concerning the evaluation of the influence of degradation phenomena on osteochondral defect repair, this work focused on mechanics-dependent tissue formation. However, the biological influence of scaffold degradation might derive also from other factors, such as local variations in pH and the release of degradation by-products (Suganuma & Alexander, 1993; Taylor et al., 1994; Sung et al., 2004). Therefore, the here presented model does not provide a complete evaluation of the complex events involved in polymeric scaffold degradation and the consequent tissue formation. Nonetheless, the model offers valuable insights on the mechanical influences that can be expected from the degradation of a synthetic polymeric scaffold with strut-like architecture in osteochondral defects. These insights could be used as indications in the selection of biodegradable or bioresorbable materials for scaffolds to support the healing of osteochondral defects. The results of this study are expected to apply also to defects of greater width implanted with a scaffold composed of the same repetitive unit. In fact, a previous *in silico* model showed similar mechanics-dependent repair patterns in untreated osteochondral defects with radii of 5 mm, 7 mm, and 9 mm, although with more fibrous tissue and less bone formation with increasing defect width (Kelly & Prendergast, 2005). On the other hand, the repair outcome supported by degradable scaffolds with non-repetitive architectural units and/or different morphologies should be specifically evaluated. Particularly, scaffolds obtained via production techniques that do not enable a precise control on the resulting material distribution might generate diverse local mechanical environments in the defect, e.g., due to walls of varying thickness or pores of different sizes. However, the simulation possibilities of the model are currently limited to scaffolds with axisymmetric geometries, thereby precluding an accurate assessment of devices with non-uniform properties in three dimensions. This issue could be addressed in future by employing a three-dimensional non-axisymmetric model.

The here-presented model could be further developed to investigate the role of scaffold-supported tissue formation also for specific subpopulations, e.g., differing in age or sex. In fact, evidence suggests that age and sex might result in differences in tissue growth, e.g., when comparing chondrogenesis and osteogenesis of MSCs seeded in decellularized extra-cellular matrix taken from new-born, juvenile, and adult rabbits (Wang et al., 2020), and in the progression of diseases, e.g., osteoarthritis (Urquhart et al., 2008). The mechanobiological rules employed to simulate tissue formation may be adapted to reproduce the experimental outcomes obtained for various groups, as previously done in the context of bone regeneration when comparing adult and elderly mice (Borgiani et al., 2019). To specifically investigate osteochondral tissue affected by osteoarthritis, additional modifications of the model, such as its geometry and the assigned material properties, might be required. In fact, osteoarthritis has been observed to influence the properties of all the joint tissues and to cause structural changes, e.g., the narrowing of the joint space (Davis et al., 2021).

**FIGURE 6:**
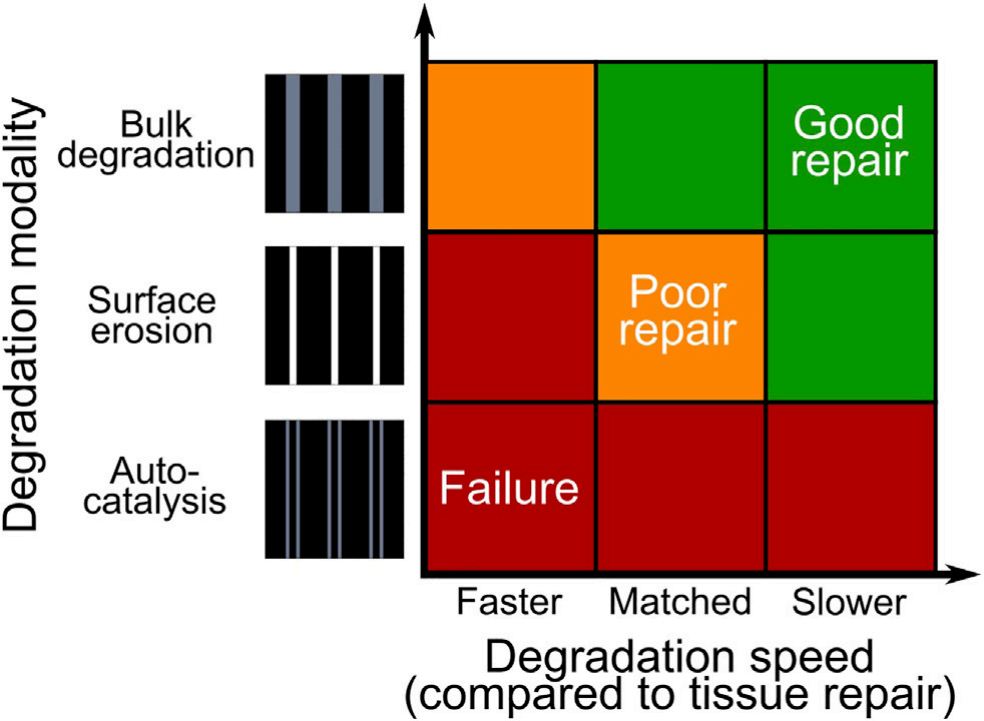
Summary of the tissue repair outcomes obtained by simulating the degradation of a synthetic polymeric scaffold with strut-like architecture implanted in an osteochondral defect. The colors red, yellow, and green indicate mechanical failure, poor repair, and good repair, respectively.

Taken together, our results suggest that, amongst the three possible degradation modalities of synthetic polymeric scaffolds with strut-like architecture, bulk degradation with autocatalysis and bulk degradation without autocatalysis cause the highest and the lowest mechanical instability of the scaffold, respectively (Figure 6). As the instauration of autocatalysis consistently resulted in the prediction of mechanical failures, we propose that the choice of material and architecture of a scaffold for osteochondral defect repair should aim at avoiding this phenomenon. Moreover, the results of the simulation indicate that scaffolds with strut-like architectures degrading by surface erosion or by bulk degradation without autocatalysis can provide an equally adequate support to the mechanics-dependent repair of osteochondral defects, but only if the scaffold material degrades in a much longer time than the time of expected completion of the repair process.

## Data Availability

The raw data supporting the conclusions of this article will be made available by the authors, without undue reservation.
