# Supplementary material for: The Degradation of Synthetic Polymeric Scaffolds With Strut-like Architecture Influences the Mechanics-dependent Repair Process of an Osteochondral Defect in Silico
Source: Front Bioeng Biotechnol. 2022 Mar 10;10:846665. doi: 10.3389/fbioe.2022.846665 (PMC8960607; doi:10.3389/fbioe.2022.846665)
Supplement: Supplementary file 1 [file DataSheet1.docx]

Supplementary Material


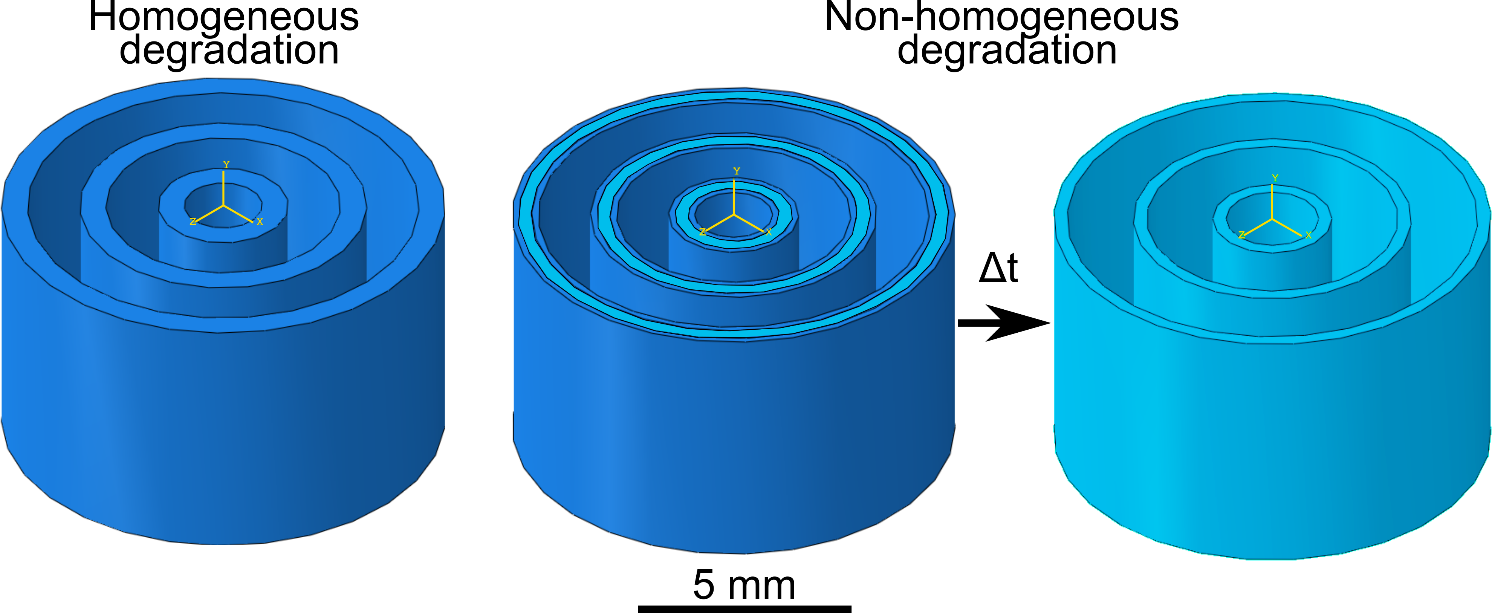


**Supplementary Figure 1.** Three-dimensional finite element model employed for the stiffness evaluation of the investigated scaffold. In the case of homogeneous degradation (left), the material properties of the scaffold were varied homogeneously. In the case of non-homogeneous degradation (middle), the scaffold was divided into surface (blue) and bulk (cyan) regions, where the material properties varied differently. When one of the two regions was completely degraded (Δt = time variation), only the remaining region was evaluated (e.g. for surface degradation, right).

**
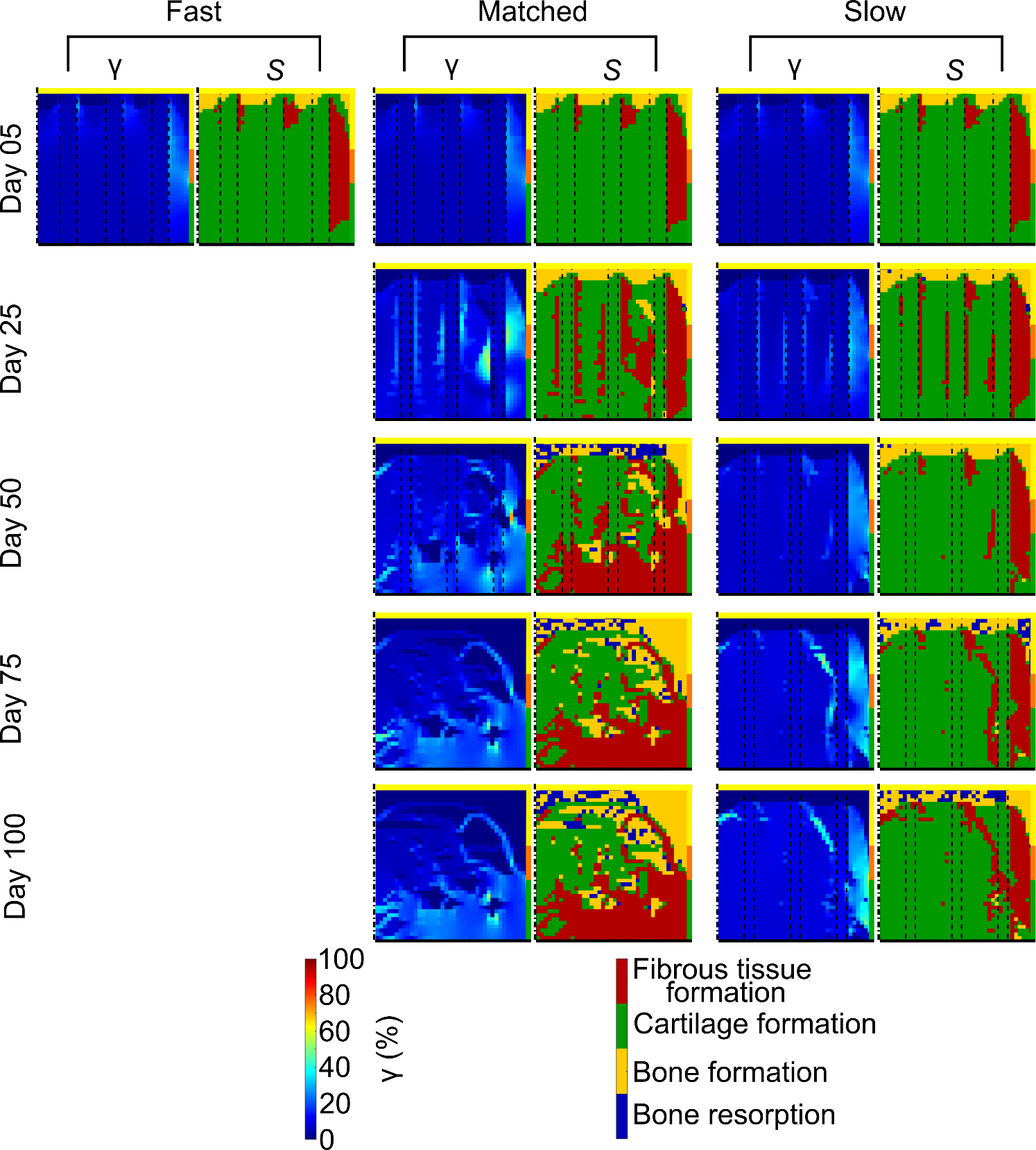
**

**Supplementary Figure 2.** Influence of scaffold degradation by surface erosion on osteochondral defect repair (prior to equilibrium). The left, middle, and right columns refer to the fast, matched, and slow degradation rates, respectively. In each column, the distribution of octahedral shear strain (*γ*) and the prediction of tissue formation based on the mechanical stimulus (*S*) are shown on the left and right side, respectively, as indicated by the captions. Each row shows a different time point, ranging from day 5 to day 100. The yellow, orange, and green borders indicate the neighboring healthy tissues of cancellous bone, subchondral bone, and cartilage, respectively. The dash-dot and solid black lines highlight the axis of symmetry and the articular surface, respectively. The scaffold struts are outlined by the black dashed lines. The legends to interpret the plots are at the bottom of the figure.


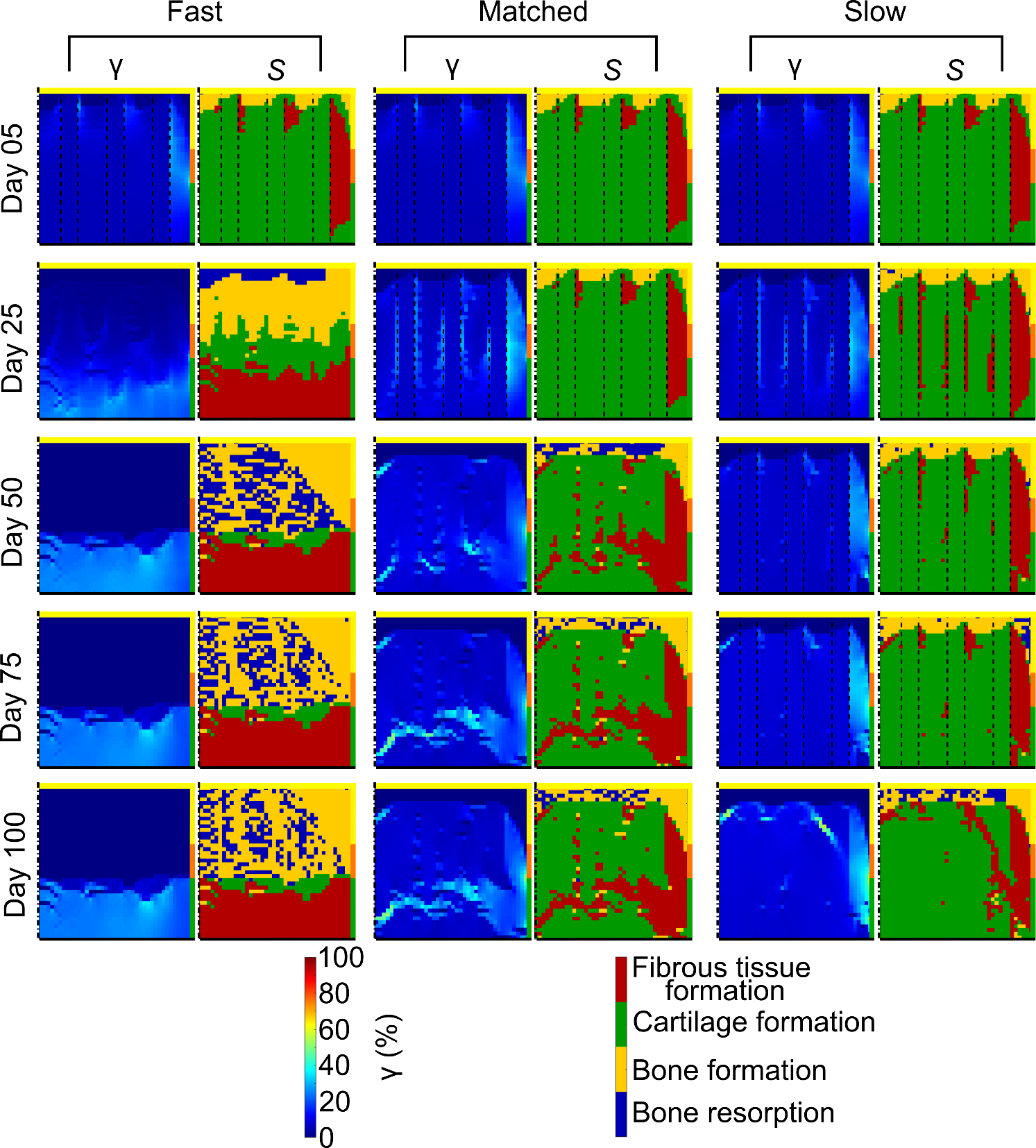


**Supplementary Figure 3.** Influence of scaffold degradation by bulk degradation on osteochondral defect repair (prior to equilibrium). The left, middle, and right columns refer to the fast, matched, and slow degradation rates, respectively. In each column, the distribution of octahedral shear strain (*γ*) and the prediction of tissue formation based on the mechanical stimulus (*S*) are shown on the left and right side, respectively, as indicated by the captions. Each row shows a different time point, ranging from day 5 to day 100. The yellow, orange, and green borders indicate the neighboring healthy tissues of cancellous bone, subchondral bone, and cartilage, respectively. The dash-dot and solid black lines highlight the axis of symmetry and the articular surface, respectively. The scaffold struts are outlined by the black dashed lines. The legends to interpret the plots are at the bottom of the figure.

**
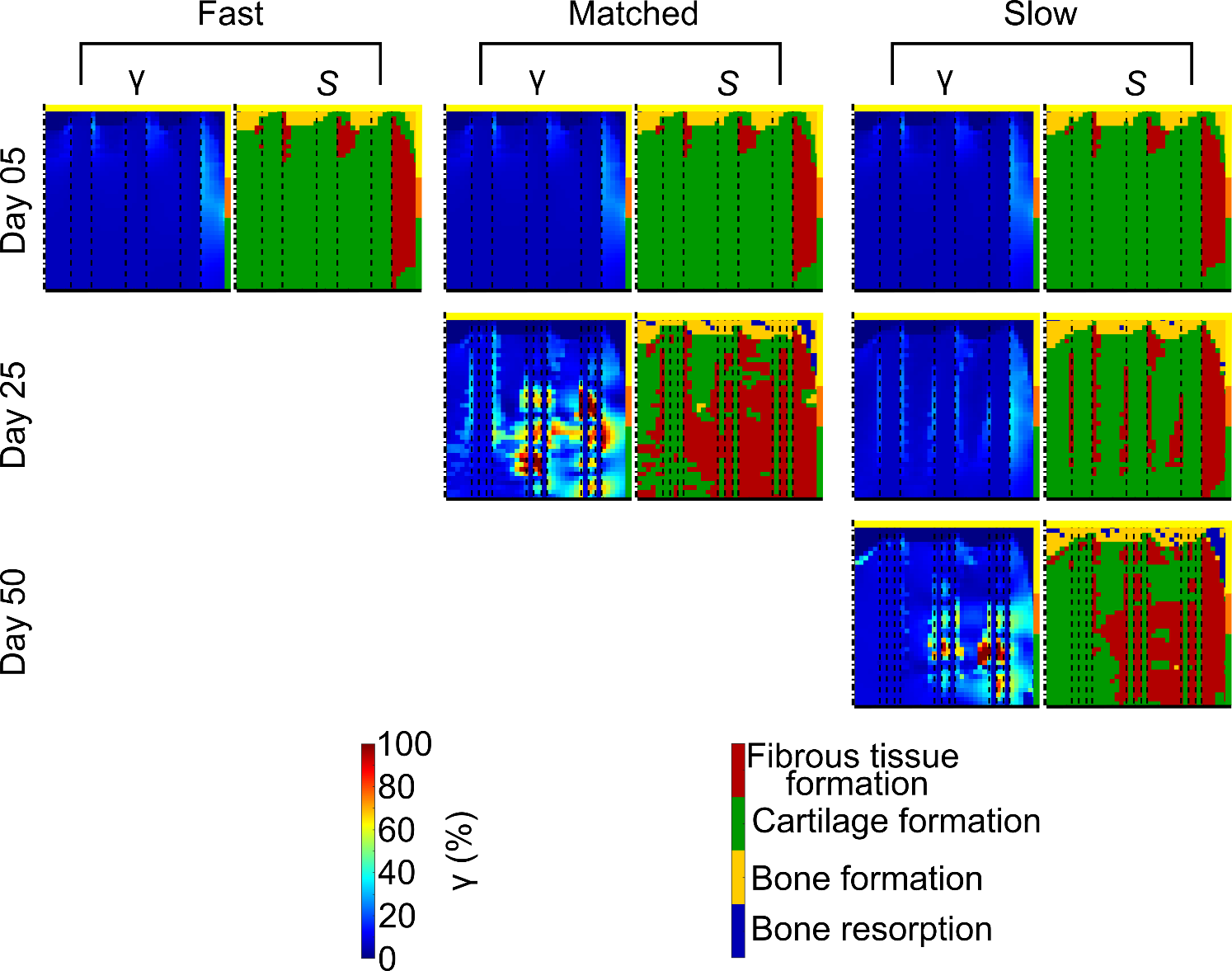
**

**Supplementary Figure 4.** Influence of scaffold degradation by bulk degradation with autocatalysis on osteochondral defect repair (prior to equilibrium). The left, middle, and right columns refer to the fast, matched, and slow degradation rates, respectively. In each column, the distribution of octahedral shear strain (*γ*) and the prediction of tissue formation based on the mechanical stimulus (*S*) are shown on the left and right side, respectively, as indicated by the captions. Each row shows a different time point, ranging from day 5 to day 50. The yellow, orange, and green borders indicate the neighboring healthy tissues of cancellous bone, subchondral bone, and cartilage, respectively. The dash-dot and solid black lines highlight the axis of symmetry and the articular surface, respectively. The scaffold struts are outlined by the black dashed lines. The legends to interpret the plots are at the bottom of the figure.


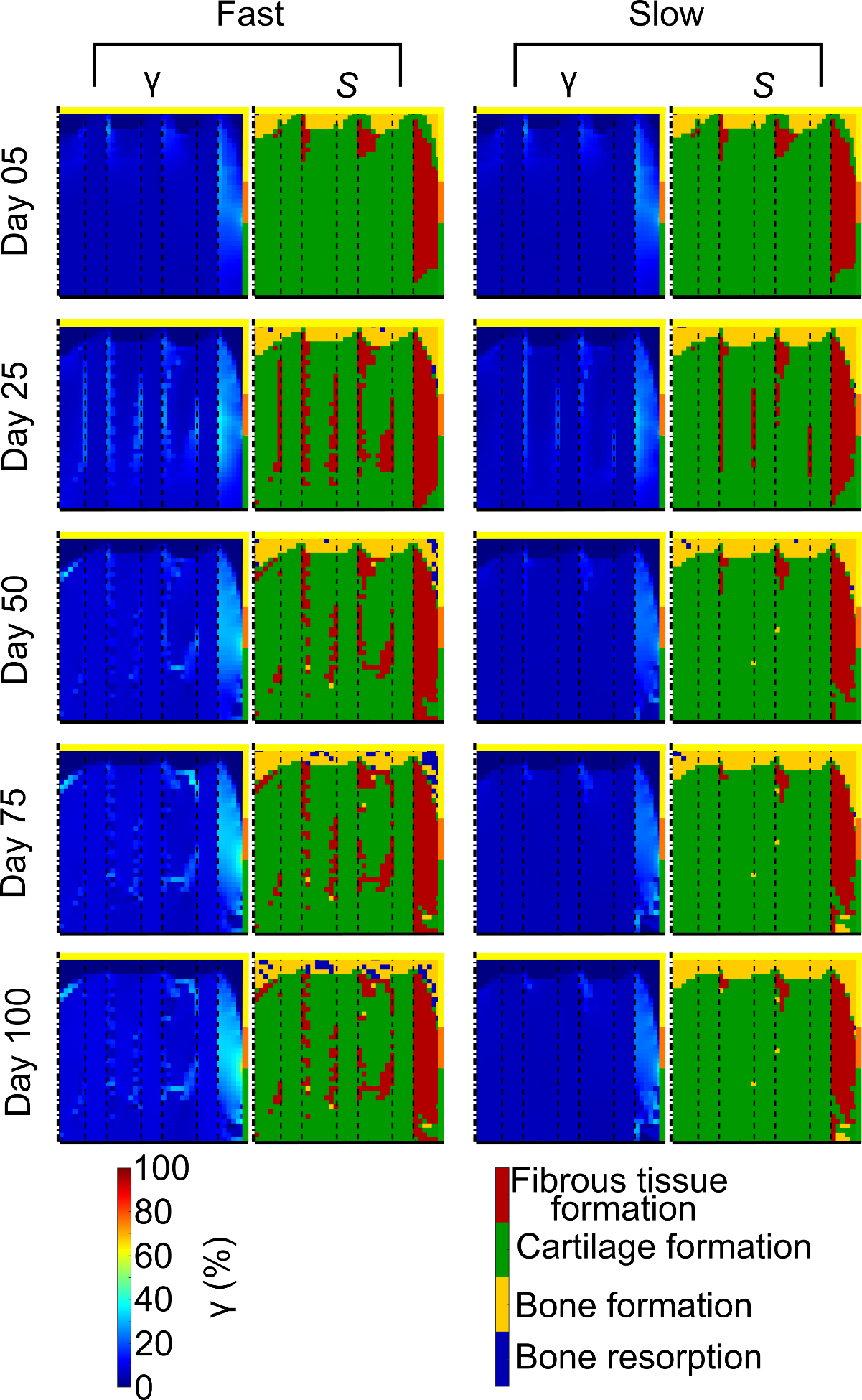


**Supplementary Figure 5.** Influence of scaffold degradation by bulk degradation based on experimental data on osteochondral defect repair (prior to equilibrium). The left and right columns refer to the fast and slow exponential degradation rates, respectively. In each column, the distribution of octahedral shear strain (*γ*) and the prediction of tissue formation based on the mechanical stimulus (*S*) are shown on the left and right side, respectively, as indicated by the captions. Each row shows a different time point, ranging from day 5 to day 100. The yellow, orange, and green borders indicate the neighboring healthy tissues of cancellous bone, subchondral bone, and cartilage, respectively. The dash-dot and solid black lines highlight the axis of symmetry and the articular surface, respectively. The scaffold struts are outlined by the black dashed lines. The legends to interpret the plots are at the bottom of the figure.
